# DeepDetectNet vs RLAttackNet: An adversarial method to improve deep learning-based static malware detection model

**DOI:** 10.1371/journal.pone.0231626

**Published:** 2020-04-23

**Authors:** Yong Fang, Yuetian Zeng, Beibei Li, Liang Liu, Lei Zhang

**Affiliations:** College of Cybersecurity, Sichuan University, Chengdu, China; Pablo de Olavide University, SPAIN

## Abstract

Deep learning methods are being increasingly widely used in static malware detection field because they can summarize the feature of malware and its variants that have never appeared before. But similar to the picture recognition model, the static malware detection model based on deep learning is also vulnerable to the interference of adversarial samples. When the input feature vectors of the malware detection model is based on static features of Windows PE (Portable Executable, PE) file, the model is vulnerable to gradient-based attacks. Regarding the issue above, a method of adversarial sample generation is proposed, which can summarize the blind spots of the original detection model. However, the existing malware adversarial sample generation method is not universal and low in generation efficiency due to the need for human control and difficulty in maintaining a normal file format. In response to these problems, this paper proposes a novel method of automatic adversarial samples generation based on deep reinforcement learning. Firstly, a static PE malware detection model based on deep learning called DeepDetectNet is constructed, the original AUC of which can reach 0.989. Then, an adversarial sample generation model based on reinforcement learning called RLAttackNet is implemented, which generates malware samples that can bypass DeepDetectNet. Finally, when we re-input the adversarial samples into the previously trained DeepDetectNet, the original defects of DeepDetectNet can be reinforced. Experimental results show that the RLAttackNet proposed in this paper can generate about 19.13% of malware samples bypass DeepDetectNet. When DeepDetectNet is retrained with these adversarial samples, the AUC value improves from 0.989 to 0.996 and attack success rate has a significant drop, from 19.13% to 3.1%, compared with the original model.

## Introduction

With human’s increasing dependence on computer system, the detection of malware has become a crucial problem in cyberspace security. According to the research of [1], a single malware event can cause millions of dollars of losses. Traditional signature-based detection methods always lag new malware or new vulnerability exploitation. Therefore, detection methods based on machine learning and deep learning are proposed.

Compared with traditional machine learning algorithms, deep learning can fit more complex nonlinear transforms. When the given amount of input data is large, a deep learning model is often able to summarize the features by itself, thus reducing the incompleteness of artificial feature extraction. Afifi et al. [2] proposed a hybrid method to find the optimum parameters that can be used to facilitate mobile malware identification. Athiwaratkun B et al. [3] constructs LSTM (long short-term memory, LSTM) and GRU (gated recurrent unit, GRU) models to detect malware. Vinayakumar R et al. [4] compared the differences between traditional machine learning algorithm (including Logistic Regression, Naive Bayes, KNN, Decision Tree, Random Forest, SVM, etc.) and deep learning algorithm when applied to static malware detection. The experimental results show that the deep learning model based on deep neural network has more advantages than traditional machine learning algorithms in the field of static malware detection.

For deep learning, in addition to the construction of the model itself, the most important work is feature extraction. But there’s no uniform standard for feature extraction in malware detection. Currently, for static PE malware detection based on deep learning, there are two main framework of feature extraction. The first one is the traditional method based on feature engineering. This method extracts features from a particular file format manually, and finally aggregates all possible features into a total feature vector, which is used as the input of the learning model. The main advantage of this method is that the extracted features are meaningful, which can parse each segment of a PE file separately. The disadvantage is that it takes a lot of work and there is no guarantee that the extracted features will be useful in practice. Starting from the PE file format, Anderson HS et al. [5] elaborates on how to extract the static features of PE files manually. Raff E et al. [6] extracts only PE headers. Sami A et al. [7] parses API (Application Programming Interface, API) calls, and Kostakis O. et al. [8] extracts function call graph as features. Awad RA et al. [9] and Nguyen MH et al. [10] extract control flow graphs. Di Xue et al. [11] combined the feature extraction methods of opcode (Operation Code, opcode) sequence and API call graph with the task of homology analysis. The main idea of the second framework is to take advantage of deep learning by letting computers extract features of a file themselves. Raff E et al. [12] proposed that PE file can be regarded as a huge byte sequence, and it can be used as input, so that the deep learning model can learn its internal relations and features by itself. Santos I et al. [13], Gandotra E et al. [14], Niu Z et al. [15], Wang C et al. [16], and Hu X et al. [17] extracts the opcode sequence of a PE file with external tools, and then takes the sequence as the input of the deep learning model, allowing the learning model to extract features automatically. Babaagba KO et al. [18] concluded the method for feature selection.

Currently, the hottest feature extraction method based on the second framework is the method called MalConv proposed in [12]. However, many researchers have since discovered the defects of doing so. Kolosnjaji B et al. [19] carried out a byte-based attack on it, which finally could generate new adversarial samples and achieve the goal of deceiving MalConv by modifying only less than 1% of bytes of the original PE file. Demetrio L et al. [20] made a further research on the basis of [19] and pointed out the exact defect of MalConv. The research of [20] found that MalConv has almost only learned some features of PE file header, which could be exploited to implement attacks. Also taking MalConv as the target of attack, Suciu O et al. [21] studied several specific methods of generating adversarial samples, the results of which also reveal the defects of the second feature extraction framework represented by MalConv.

Therefore, the static PE malware detection model based on deep learning called DeepDetectNet is constructed in this paper, which uses the traditional feature extraction method based on feature engineering. Having summarized the existing feature extraction methods based on feature engineering, this paper proposes a new feature extraction method. The deep learning model using this method can achieve 0.989 of AUC with around 7000 samples for training and testing. The experimental results show that the AUC value of DeepDetectNet is slightly better than 0.986, the AUC value of the method proposed in [5].

**Fig 1:**
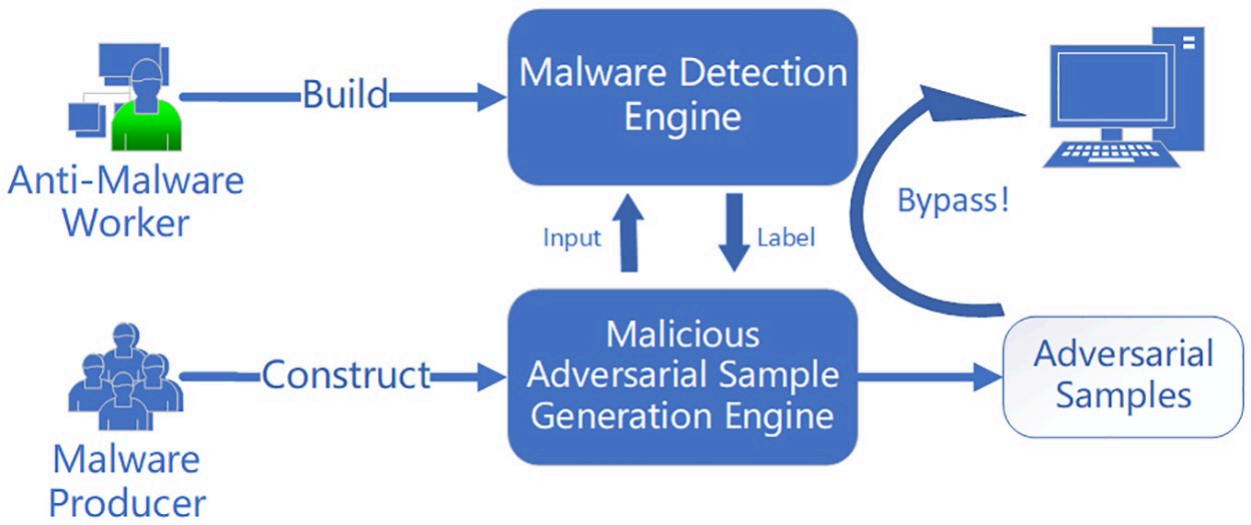
The threat of adversarial samples. Attackers can produce malicious adversarial samples that can cheat the malware detection engine.

However, as shown in Fig 1, there are always some defects in a static malware detection model based on deep learning, which cannot be directly proved by complex mathematical formulas. In order to find out the defects of DeepDetectNet, we need to explore this issue from the perspective of an attacker. By generating adversarial samples, we can discover the defects that are difficult to be found directly in DeepDetectNet.

The existing attack methods against machine learning and deep learning detection model are mainly divided into black box attack and white box attack [22]. Octavian Suciu et al. [23] investigated some existing strategies for adversarial example generation against static malware detection model based on CNN (Convolutional Neural Network, CNN), including *Append*—*based Attacks* and *Slack*—*based Attacks*. The experiments indicate that the effectiveness of adversarial attacks on models trained using small datasets does not always generalize to robust models. The works of [19, 20], and [23] are representations of white box attack on deep learning. In [24], a black box attack method based on reinforcement learning is proposed. Fang Z et al. [25] improved the *Reward* method in [24] and obtained a higher success attack rate. Both methods proposed in [24] and [25] are black box attack on traditional machine learning. But the experimental results show that adversarial samples generated by the method proposed in [25] are all UPX packed because of some thoughtlessness with the method of modifying PE files.

Considering the problems above, the method of modifying PE files is improved and an attacking model with the framework of double and dueling DQN (Double Q-Network, DQN), called RLAttackNet, is proposed. We use RLAttackNet built by this newly improved method to implement a black box attack on DeepDetectNet. The attack success rate can reach 19.13%.

Finally, inspired by GAN [26], we re-use the generated effective adversarial samples as the input and retrain DeepDetectNet. The AUC of DeepDetectNet could improve to 0.996. The bypass rate itself (calculated by Eq (8)) dropped from 1.93% to 0.83%. Moreover, using RLAttackNet again to attack the retrained DeepDetectNet, the attack success rate dropped from 19.13% to 3.1%.

In conclusion, the main contributions of this paper are summarized as follows:

- **Feature Engineering**
  On the basis of existing research about feature extraction based on feature engineering, we proposed a novel method for extracting PE file features. We further studied and improved the import function features and streamlined some unnecessary features. In the end, our method of feature engineering consists of three aspects, including Import Function Feature, General Information Feature, and Bytes Entropy Feature.
- **Adversarial Sample Generation Based on Reinforcement Learning**
  We proposed a novel framework for generating adversarial samples based on reinforcement learning. For the *Agent*, we built the double and dueling DQN architecture. Moreover, we built the *Action Space* that do not break the structure and functions of a PE file, while each action taken by *Agent* is certain.
- **Retrain Detection Model Using the Idea of GAN**
  Drawing on the idea of game theory in GAN, we retrained our malware detection model with newly generated adversarial samples. Surprisingly, the performance of retrained detection model is extremely good.

## Related work

### Main thought of GAN

In 2014, Goodfellow et al. [26] proposed a deep learning model called GAN (Generative Adversarial Network, GAN). The model is a fitting problem of probability distribution in mathematical principle, but the original paper emphasized the idea of game theory. In a GAN system, there is a deep neural network called Generator G and another deep neural network called Discriminator D. Take image generation as an example. The input of generator G is a random high dimensional vector, and the output is another high dimensional vector. When the value of each dimension of the output vector is mapped to each pixel of the image, the output can be regarded as an image. The input of discriminator D is an image. More specifically, each pixel is treated as the value of a vector for each dimension, and the input is also a vector, which has the same dimension as the output vector of generation G. The output of discriminator D is a scalar, and the larger the value is, the more likely the input image is real. The lower the value, the more likely the input image is fake. The idea can be expressed by mathematical expression as shown in Eq (1).

$$\begin{array}{c} \begin{array}{cc} \underset{G}{\text{max}}\underset{D}{\text{max}}\;V(D,G) & =E_{x∼P_{data}\left(x\right)}\left[logD\left(x\right)\right]\\ & +E_{z∼P_{z}\left(z\right)}\left[log\left(1-D\left(G\left(z\right)\right)\right)\right] \end{array} \end{array}$$ (1)

In Eq (1), *V*(⋅) is a value function. If input x is the data from real samples, the output value of discriminator *D* should be as large as possible. If the input comes from the data *G*(*z*) generated by generator *G*, the output value of discriminator *D* should be as small as possible. Initially, both generator *G* and discriminator *D* performed poorly. In the process of iteration training, the generator *G* keeps deceiving the discriminator *D* of the previous iteration, and the discriminator *D* keeps identifying samples generated by generation *G* of the previous iteration. After multiple iterations, both the performances of generator *G* and discriminator *D* are significantly improved.

In GAN, the relationship between generator *G* and discriminator *D* can be seen as both antagonistic and mutually reinforcing.

In the field of static malware detection, methods based on the idea of GAN have already made some breakthrough. For example, Vega-Márquez et al. [27] has focused on the creation of new synthetic data from the “Default of Credit Card Clients” dataset with a Conditional Generative Adversarial Network (CGAN), the results of which show that GAN-based methods have great potential in synthesizing new data. Kim et al. [28] proposed a novel method called transferred deep-convolutional generative adversarial network (tDCGAN), which generates fake malware and learns to distinguish it from real malware. The results show the promising ability of GAN-based method to detect zero-day malware.

Although the algorithm is not directly applied in this paper, its idea of game theory is used in the design of the overall architecture.

### Black-box attack method based on reinforcement learning

In order to simulate the real scenario, when attacking the static PE malware detection model, the common attack mode is black-box attack. In a black-box scenario, the attacker does not know any detail of the detection model, which can simulate a more realistic attack condition. In the context of black-box attack, reinforcement learning-based methods have shown great potential. Zhao et al. [29] proposed the feature-interference reinforcement (FIR) method with the enhanced realistic constraints generation (ERG), which is used to enhance robustness, to generate adversarial samples (AEs). The AEs show the capability of attacking three state-of-the-art black-box models with high success rate. Tsingenopoulos et al. [30] introduced AutoAttacker, a novel reinforcement learning framework for black-box adversarial attacks. The experimental evaluations were carried out with the black-box MNIST classification model, the results of which demonstrate the practical feasibility of reinforcement learning-based black-box attack approach. The work of Hyrum S. Anderson et al. [24] in 2017 proposed a creative method that attacks a machine-learning malware detection model based on reinforcement learning. In their work, the *Action Space* which is able to modify a PE file without breaking it was build. Their model supports byte streams of a PE file as input and the success attack rate can reach 16%.

In our work, our attack target become deep neural network and we improved their method by several aspects. Firstly, we modified the interface so that our Agent can receive a file name as input, which makes debugging easier. Secondly, we redesigned the *Action Space*, eliminating the randomness when modifying a PE file. Thirdly, we improved the *Agent* in reinforcement learning by constructing it with double dueling DQN. Last but not least, we improved the method to calculate the *Reward* in an episode.

### Reinforcement learning and Q-learning

**Fig 2:**
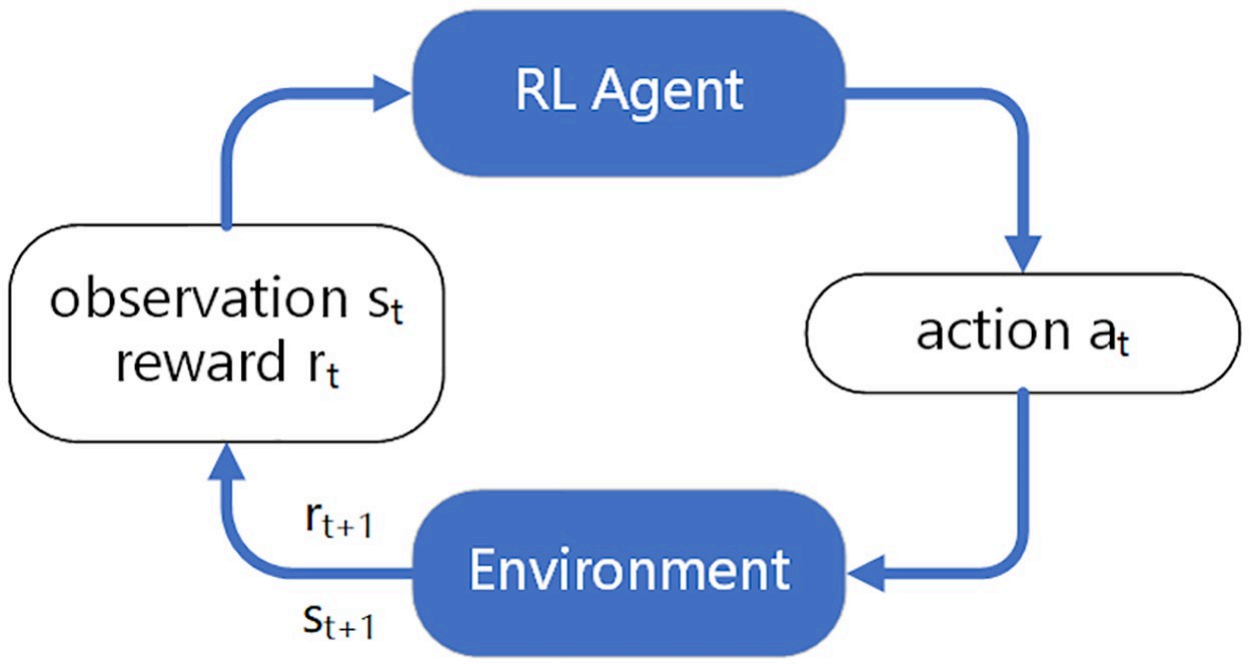
A reinforcement learning scenario.

Reinforcement learning belongs to the category of machine learning and is another learning algorithm besides supervised learning and unsupervised learning. In a reinforcement learning system, there are mainly four elements: an *Agent* for learning, the current *Environment State*, the *Action* *Space* in which an *Agent* can take an *Action*, and the Reward that an *Agent* can eventually obtain. What an *Agent* needs to learn is to select the best *Action* in a given *Environment* *State* so as to maximize the Reward he can obtain. A general reinforcement learning scenario is shown in Fig 2.

Several reinforcement learning-based methods have been proposed in the field of static malware detection. For instance, Fang et al. [31] proposed an architecture using reinforcement learning to implement feature selection task. Experiments show that the architecture can discover features for malware detection. Wan et al. [32] have formulated a cloud-based mobile malware detection method based on Deep Q-learning (DQN). The DQN-based malware detection method accelerates the learning speed, increases the malware detection accuracy, reduces the detection delay, and improve the utility.

**Fig 3:**
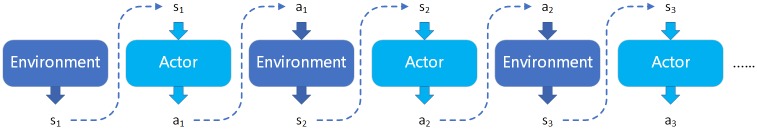
The process of a trajectory.

The reinforcement learning model based on policy-gradient is the basic model of reinforcement learning. Take Atari [33] game as an example. In this game, a player controls the plane below, and each step has three choices: move left, move right, and fire. There will be a *reward* In this paper, a lowercase *reward* represents a “reward” once, and the *Reward* in uppercase represents the accumulated “Reward” after a trajectory. when a monster is killed. In a complete game, the final *Reward* is the sum of each *reward*. Each game image can be considered a *Environment State*, represented by {*s*_*t*_}(*t* = 1, 2, …, *n*). Under a certain *State* *s*_*t*_, the *Action* taken by the *Agent* is represented as *a*_*t*_, the process of which is shown in Fig 3.

Let

$$\begin{array}{c} \text{Trajectory}\;\tau =\{s_{1},a_{1},s_{2},a_{2},\ldots ,s_{T},a_{T}\} \end{array}$$ (2)

Having played several rounds of game, the *Agent* can get many groups of *τ*, and the sequences in each group of *τ* are different. Based on these obtained *τ*, the *Agent* can calculate the frequency of a certain *τ* under the control of parameter *θ*, and regard the frequency as the probability of the occurrence of *τ*:

$$\begin{array}{c} p_{\theta }(\tau )=p(s_{1})p_{\theta }(a_{1}|s_{1})p(s_{2}|s_{1},a_{1})p_{\theta }(a_{2}|s_{2})p(s_{3}|s_{2},a_{2})\ldots \end{array}$$ (3)

where *p*(⋅) represents the probability controlled by the *Environment* itself, independent of the parameter *θ*.

If the *Agent* is different, then for the same *s*_*t*_, its *Action* *a*_*t*_ is likely to be different. For a reinforcement learning model, another crucial part is the *Reward*. In the simplest case, a *Reward* is the sum of all the rewards that have been obtained after a **Trajectory** *τ*, expressed as Eq (4):

$$\begin{array}{c} R(\tau )=\sum_{t=1}^{T}r_{t} \end{array}$$ (4)

where *r*_*t*_ represents the *reward* obtained after taking the *Action* *a*_*t*_ at *s*_*t*_. For the entire learning process, the *Reward* is no longer a specific value, but a random variable. Therefore, for the whole model, the *Reward* should be an expectation relative to parameter *θ*, expressed as Eq 5.

$$\begin{array}{c} \bar{R_{\theta }}=\sum_{\tau }R(\tau )p_{\theta }(\tau )=E_{\tau ∼p_{\theta }(\tau )}[R(\tau )] \end{array}$$ (5)

According to Eq (5), if the parameter *θ* is fixed, the probability of the occurrence of a certain *τ* and its corresponding *R*(*τ*) is computable, so that the expectation of *Reward* of the entire model is also computable. The goal of learning is to make the expectation of *Reward* as large as possible by updating the parameter *θ*.

Policy gradient is the algorithm designed to update parameter *θ* for this goal. Similar to the gradient descent method in deep learning, the update expression for parameter *θ* is presented by Eq (6).

$$\begin{array}{c} \theta \leftarrow \theta +\eta \nabla \bar{R_{\theta }} \end{array}$$ (6)

According to the above Eqs (3), (4) and (5), it can be proved that $\nabla \bar{R_{\theta }}$ in Eq (6) is computable. Therefore, the reinforcement learning model can converge to a satisfying training result.

In Fig 3, the *Actor* interacting with the environment is the same as the *Agent* to be learned. This learning mode is called **on-policy** mode. In contrast, the learning mode that the *Agent* is different from the *Actor* is called **off-policy** mode.

Q-learning is an off-policy method, in which a new element called *Critic* needs to be defined in the model. Rather than directly deciding what *Action* to take, the *Critic* evaluates an *Actor* *π*. Firstly, we define a function *V*^*π*^(*s*) named *Value Function*, which refers to the expectation of the rest accumulated *Reward* when the *Environment State* is s, using *π* as the *Actor*. The output of this function is obtained according to the statistics of *π*. Another function *Q*^*π*^(*s*, *a*) called *Q* − *function* is defined here, which means the expectation of the accumulated *Reward* obtained by using *π* as the *Actor* after forcing *a* as the *Action* in the *Environment State* *s*. With *Q* − *function*, the *Agent* can decide which *Action* to take.

The update mechanism of Q-learning is expressed by Eq (7):

$$\begin{array}{c} \begin{array}{cc} Q^{\pi }(s_{t},a_{t}) & \leftarrow (1-\alpha )\cdot Q^{\pi }(s_{t},a_{t})\\ & +\alpha \cdot (r_{t}+\gamma \cdot \underset{a}{\text{max}}\;Q^{\pi }(s_{t+1},a)) \end{array} \end{array}$$ (7)

where *α* denotes the learning rate, and *γ* denotes the discount factor, indicating the influence of future *reward* on the current *Action*. In Eq (7), the most important item is $\max_{a}Q^{\pi }(s_{t+1},a)$. Let

$$\begin{array}{c} \pi^{\prime }(s)=arg\underset{a}{\text{max}}\;Q^{\pi }(s,a) \end{array}$$ (8)

It can be proved that $V^{\pi^{\prime }}(s)\geq V^{\pi }(s)$ always holds for all States *s*. Therefore, the process of parameter updating is valid.

## Method

Drawing on the idea of GAN, we will build a static PE malware detection model called DeepDetectNet and an adversarial sample generation model called RLAttackNet, simulating the discriminator and generator in GAN respectively. Different from GAN, the input of RLAttackNet is not random noise, but a malicious PE file. The goal of DeepDetectNet is to distinguish malicious samples from benign samples, while the goal of RLAttackNet is to disguise malicious samples as benign samples. In this paper, label “1” represents malicious, and label “0” represents benign.

**Algorithm 1** Training Algorithm of DeepDetectNet

1: Extract features from original training file set and label each feature vector

2: Build the deep neural network with optimize function *Adam* and loss function *binary*_*crossentropy*

3: Fit the training set batch by batch

4: Fix the parameters of DeepDetectNet *θ*_*D*_

5: Generate malicious adversarial samples *MAS* against DeepDetectNet (with parameters *θ*_*D*_) using RLAttackNet

6: Add *MAS* to training file set

7: Retrain DeepDetectNet with new training file set

**Fig 4:**
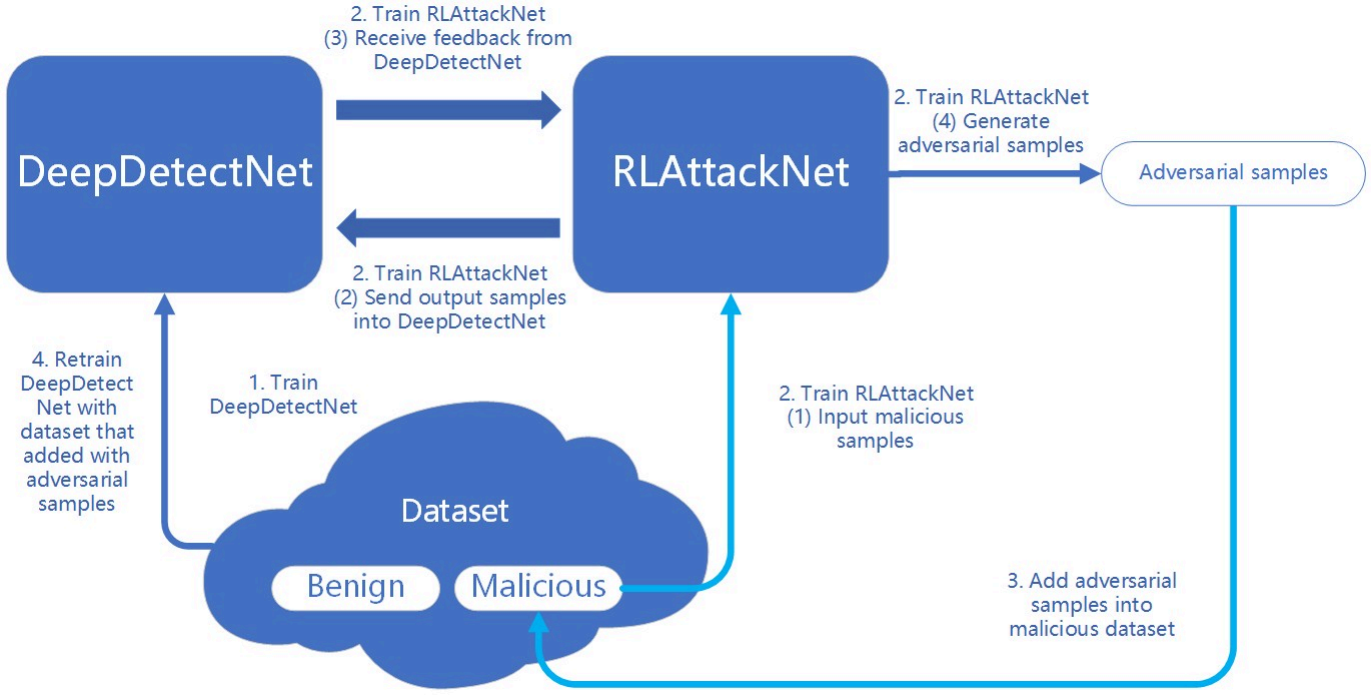
An overview of our architecture.

The main framework of training DeepDetectNet is summarized in Algorithm 1 and an overview of our architecture is shown in Fig 4. Firstly, we will construct a deep neural network called DeepDetectNet as the static PE malware detection engine. Next, we will build a deep Q-network with double and dueling architecture called RLAttackNet, which will concentrate on generating malicious adversarial samples. Finally, we will add the adversarial samples into original dataset and retrain DeepDetectNet, observing if the performance of DeepDetectNet improves.

### Construction of DeepDetectNet

This work mainly includes two steps, one is static feature extraction of PE files, the other is the construction of deep neural network.

According to [12] [19] [20] and [21], the second framework of static PE feature extraction methods mentioned in, including byte sequences method and opcode method, is still vulnerable. So, the static PE feature extraction method of this paper is based on feature engineering.

#### Static feature extraction of PE files based on feature engineering

**Fig 5:**
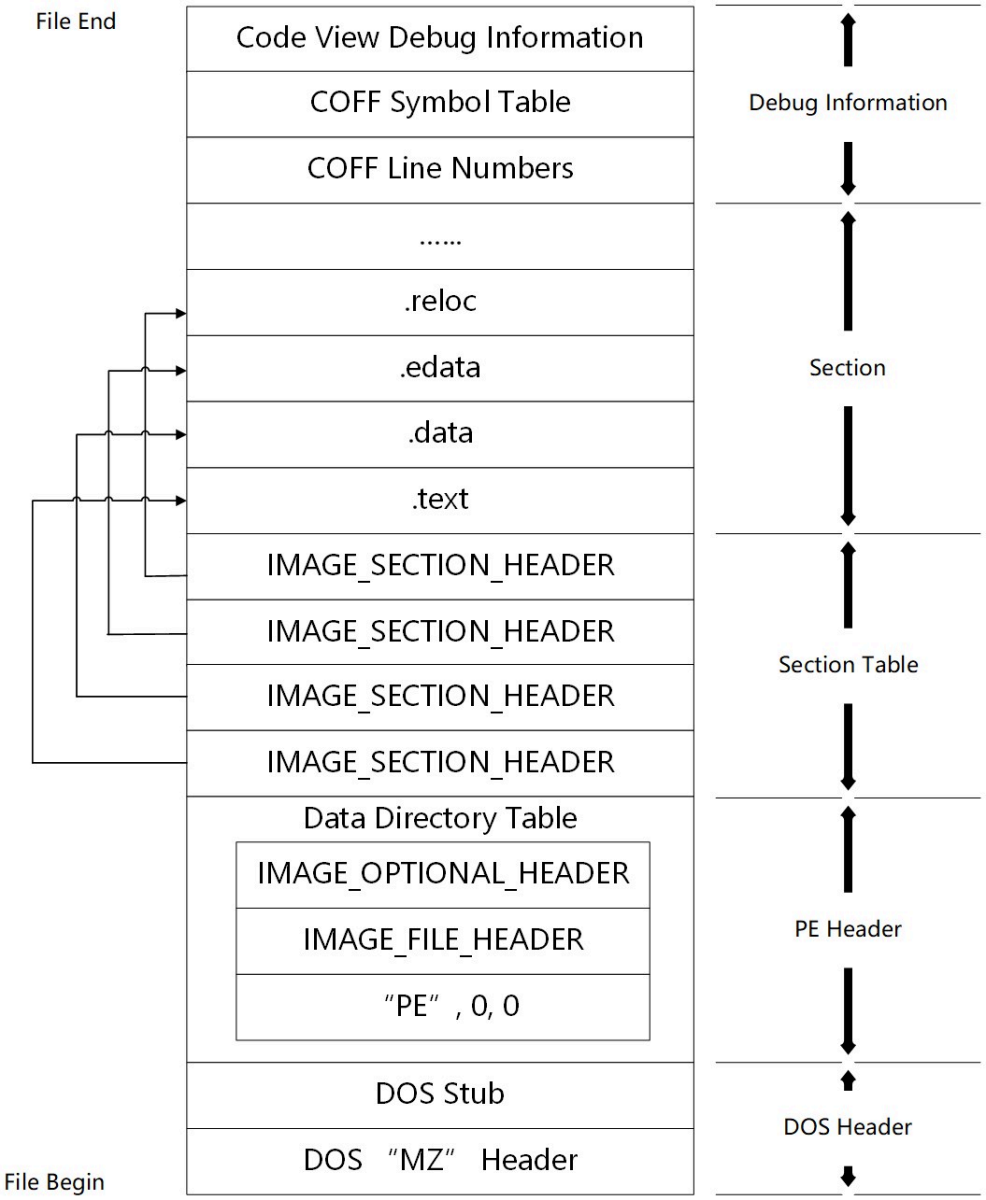
PE file format.

PE file format is an abbreviation for Portable Executable File Format under Windows. Common executables (.exe files) and dynamic link libraries (.dll files) are PE files. The format of PE file is shown in Fig 5.

From the file header to the end of the file, a PE file is composed of: DOS Header, PE Header, Section Table, Section and Debug Information. All PE files are organized in this format strictly. When modifying a PE file directly, the modification should also follow this format strictly.

The PE Header contains the starting relative virtual addresses of import table, export table and resource directory. The import table records the dynamic link library (DLL) and functions to be invoked by the PE file. Functions in the import table are called import functions. Similarly, the export table records the functions that the file will export, and we call the functions in the export table export functions.

The section table contains the names of each section, such as .text section and .data section. The section table records the relative virtual addresses of sections and some important information about them.

In general, the PE files are organized like a dictionary, the headers of which provide a summary of information about the file’s contents.

When it comes to features of PE files, there are mainly two types of features that can be extracted. The first type of features is those related to the PE file format, such as PE Header Information, Import Function Information, Section Information, etc. The second type of features is independent of file format, such as Bytes Entropy.

In theory, for the first type of features, every part of a PE file can be used to extract features. However, after experiments, we found that not every part of a PE file is valuable for deep neural network. Therefore, the static feature extraction method in this paper mainly consists of three categories, including Import Functions Feature, General Information Feature and Bytes Entropy Feature.

For Import Functions Feature, this paper maintains a list of 2212 functions, each of which represents a common import function. For each input PE file, there is a 2212-dimension feature vector corresponding to the list. In the initial state, each value in the vector is 0, meaning that there is no any import function. When parsing a PE file, if a common import function from the list appears in the import function table of the file, the corresponding position of the function in the vector is set to 1, and the other positions are still 0.

For example, if a PE file contains 98 import functions, 79 of which are in the list of common import functions, then its corresponding import function feature vector should have 79 positions set to one, and the rest of the positions should be 0. In general, most values of the 2212-dimension import functions feature vector are set to 0, while a few values are set to 1.

For General Information Feature, focusing on the profile of the PE file, we extracted 10 features that can describe the overall attributes of a PE file, so the dimension of this feature vector is 10, as shown in Table 1. The size component represents the total length of bytes of a PE file, while the *vsize* component represents the virtual size of a PE file. The *has*_*debug*, *has*_*relocations*, *has*_*resources*, *has*_*signature*, *has*_*tls* are all integers that show if a PE file has corresponding information to these variable names. The exports and imports variables represent the length of export table and import table of a PE file respectively. The symbol variable corresponds to the length of the symbol table of a PE file.

**Table 1:** General information features.

Name	Type
*size*	int
*vsize*	int
*has*_*debug*	int
*exports*_*len*	int
*imports*_*len*	int
*has*_*relocations*	int
*has*_*resources*	int
*has*_*signature*	int
*has*_*tls*	int
*symbols*_*len*	int

Bytes Entropy Feature is the feature extraction method independent of file format proposed in [34] in 2015. This method has nothing to do with PE file format. The method slides a 1024-byte window over an input binary, with a step size of 256 bytes. For each window, it computes the base-2 entropy of the window, which can be expressed by mathematical expression as shown in Eq (9).

$$\begin{array}{c} H=-\sum_{i}P_{i}logP_{i} \end{array}$$ (9)

Where the base of the logarithm is 2. *P*_*i*_ is the frequency of a byte appearing in a window. Each individual byte occurrence in the window with this computed entropy value is stored in 1024 pairs in a list. Finally, it computes a two-dimensional histogram over the pair list, where the histogram entropy axis has sixteen evenly sized bins over the range [0, 8], and the byte axis has sixteen evenly sized bins over the range [0, 255]. Having concatenated each row vector in this histogram into a single, 256-value vector, a 256-dimension feature vector is obtained.

From what has been discussed above, the dimension of our input feature vector is 2212 + 10 + 256 = 2478.

#### Deep neural network structure of DeepDetectNet

According to the research of [4], in the field of static malware detection, deep neural network has more advantages than shadow neural network with traditional machine learning algorithm. Therefore, the static PE malware detection model in this paper uses the deep neural network architecture.

**Fig 6:**
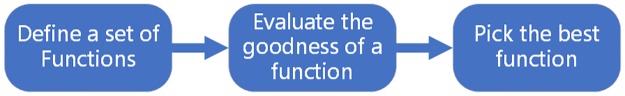
General three steps of deep learning.

Deep learning is a process of updating parameters under a specific structure of a deep neural network. For a single neuron, the parameters are the weights *w* and biases *θ*. For the whole deep neural network, its learning process is the process of updating the parameters of all neurons. When the parameters do not change any more, or they get convergence, the learning process is complete. As shown in Fig 6, the general process of deep learning can be roughly decomposed into three steps.

- Define a set of function.
  It is a processing of constructing the structure of a deep neural network. In deep learning, the number of layers of neural network, the number of neurons in each layer, and the connection mode should be decided by human. Besides, the values of hyper-parameters in the deep neural network can highly influence on the results [35]. So the hyper-parameter optimization method is also important.
- Evaluate the goodness of function.
  This is equivalent to evaluating the current parameters in a deep neural network structure. For this, the concept of loss function needs to be introduced. The loss function is a function that evaluates how much the current output differs from the actual output, and the larger the value, the worse the parameters of the deep learning model are. So, in the process of training, the neural network needs to learn how to reduce the loss. Common loss functions include Mean Square Error (MSE) and Cross Entropy.
- Pick the best function.
  This step is the process of reducing the loss, or the process of updating parameters. In deep learning, the theoretical basis of loss reduction is gradient descent. In practice, there are several parameter updating methods based on gradient descent, such as *Momentum* [36], *RMSProp* [37], *Adam* [38] and so on.

We will build the deep learning model step by step.

Considering the large dimension of input feature vector, the number of neurons in each hidden layer of our neural network should not be too small. Moreover, in the feature vector corresponding to the import function, most positions have little influence on the next layer, so the number of neurons in each hidden layer should not be too large, either. According to the experimental results, when the number of neurons in each layer is fixed at 999, we can obtain a satisfying result. To ensure that the variance of the input data is not too large, there should be a layer of batch normalization [39] in front of each layer except input layer. The connections between neurons are directly in the form of full connection.

Since the values of hyper-parameters in the deep neural network can highly influence on the results, we apply the random search method proposed in [40] to optimize hyper-parameters. This optimization method, which has been applied in several work, can find better models by effectively searching a larger, less promising configuration space. For example, the work of [35] applied random search method to optimize the hyper-parameters of their deep neural network for power consumption forecasting task, the results of which show that a random search produces competitive accuracy results generating a smaller number of models, and the smoothing process reduces the forecasting error.

**Fig 7:**
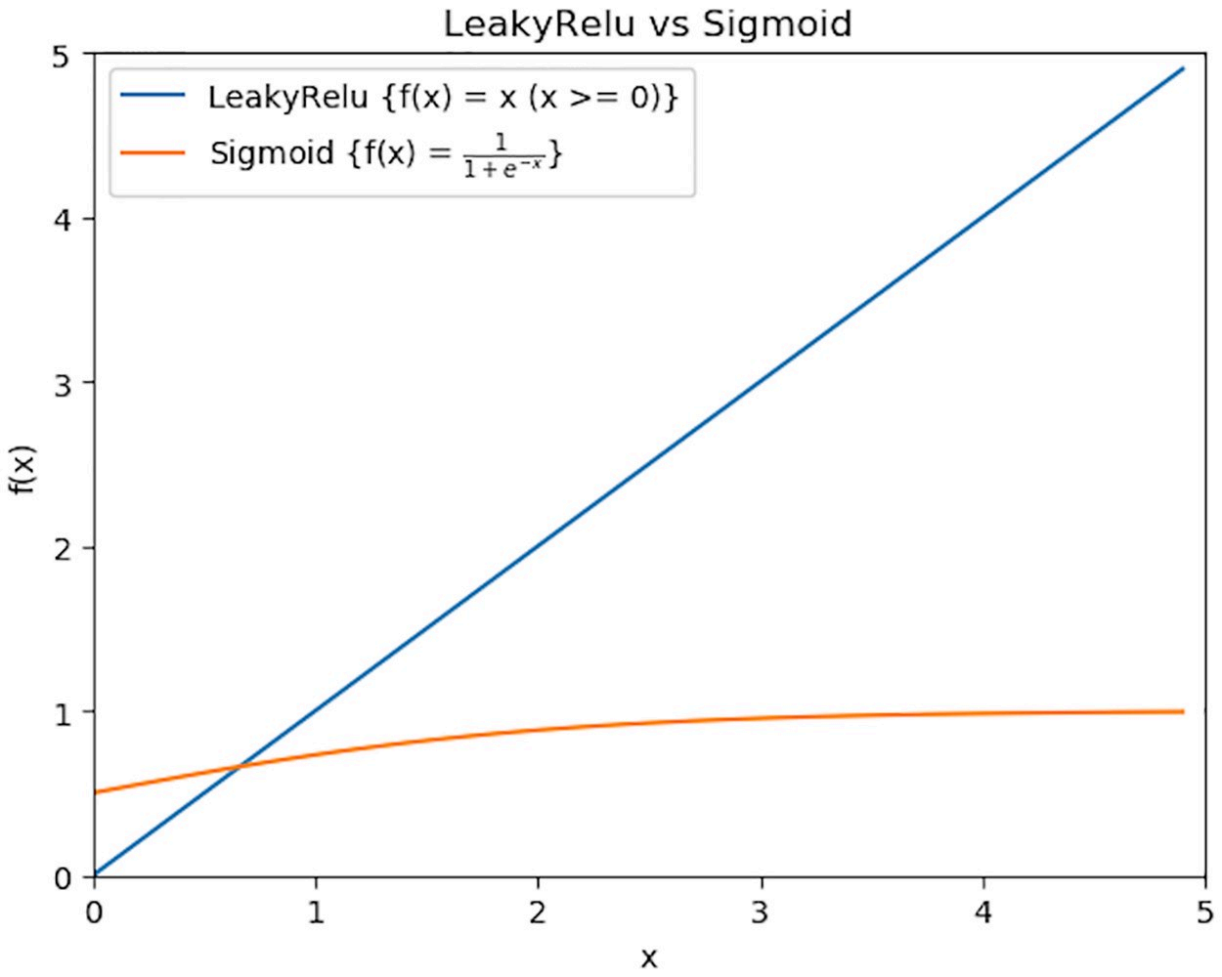
*LeakyRelu* (blue line) vs *sigmoid* (orange line) when x is greater than 0. When the neural network is deep and if *sigmoid* is deployed as the activation function in middle layers, the contribution of neurons in the front layers will become smaller and smaller to the whole neural network. However, *LeakyRelu* can keep us away from this problem.

**Fig 8:**
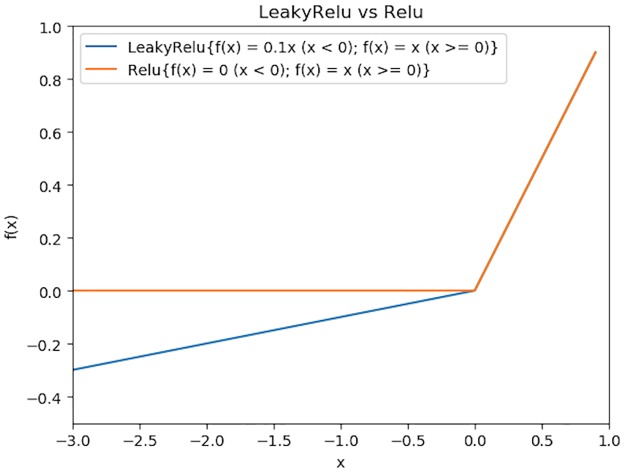
*LeakyRelu* (blue line) vs *Relu* (orange line) when x is less than 0. Unlike *Relu*, *LeakyRelu* ensures that there is still a gradient when the neuron output is negative, so that the neuron does not “die”.

Another crucial component of the deep neural network’s structure is the activation function. In our deep neural network architecture, we selected *LeakyRelu* as the activation function in the hidden layers. There are two reasons for this. Firstly, some activation functions like *sigmoid* have a decreasing derivative when the input is greater than 0, resulting in less and less influence of the front neuron on the output. If the neural network is deep, then the neurons in the first layers will have little effect on the output, which makes the deep neural network meaningless. Different from this type of activation functions, *LeakyRelu* ensures that the derivative remains constant when the input is greater than 0, allowing the neurons in the front to have the same effect on the output, as shown in Fig 7. What’s more, *Relu* function also has the same characteristics, but when a neuron is not active, its gradient is 0, so that the inactive neuron cannot update the weight value through the gradient descent method. By comparison, *LeakyRelu* allows a small gradient when the neuron is not active, so that the inactive neurons can also keep updating. The comparison between *Relu* and *LeakyRelu* is shown in Fig 8. Based on the two reasons above, *LeakyRelu* is selected as the activation function of the hidden layers, with *α* value 0.2. Since the deep neural network is to solve the binary classification problem, the activation function of the output layer is selected as *sigmoid*.

The second step is to evaluate the goodness of a function, which is equal to select a suitable loss function. In the binary classification problem, the loss function is usually selected as *binary*_*crossentropy*, which matches the *sigmoid* function of the output layer, as shown in Eq (10).

$$\begin{array}{c} C=-\frac{1}{N}\sum_{i=1}^{N}y_{i}\cdot \text{log}\;(p(y_{i}))+(1-y_{i})\cdot \text{log}\;(1-p(y_{i})) \end{array}$$ (10)

In Eq (10), *y* is the label and *p*(*y*) is the predicted label, and *N* is the total number of training samples.

The third step is to pick the best function, which is equal to select a suitable optimizer. Traditional gradient descent based optimizers, like *SGD*, are easy to fall into local optimum. *Momentum*, *RMSProp* and *Adam* algorithms simulate the inertia of an object as it moves, which reduce the probability of falling into local optimum. Comparative experiment results, as shown in Table 2, tell us that the deep learning model performs best when using *Adam* as the optimizer, with the learning rate 0.0005, the iteration epochs 20, and the batch size 100.

**Table 2:** Comparison of different optimizers.

	*Momentum*	*RMSProp*	*Adam*
**AUC**	0.967	0.977	0.989

In addition, a batch normalization layer is added in front of each activation function layer to ensure that the data variance is not too large, which would affect the process of weight change.

**Fig 9:**
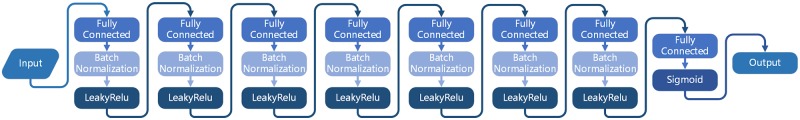
The detail of each layer.

**Fig 10:**
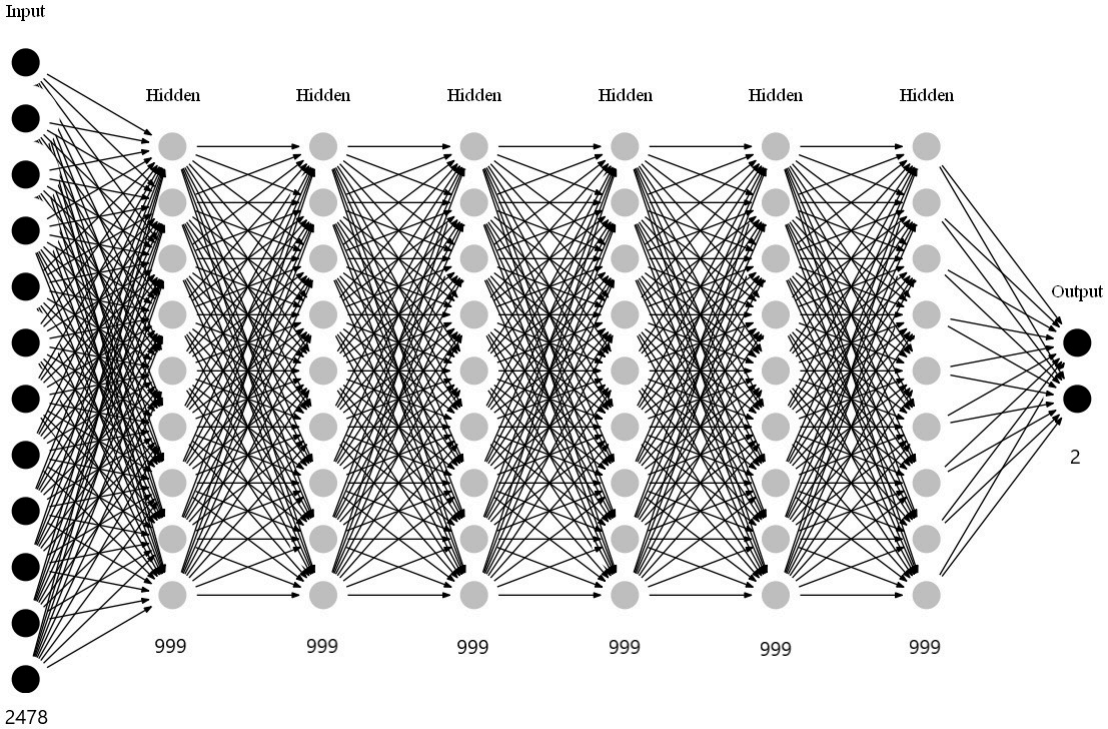
Overall structure of our neural network.

Thus, the construction of DeepDetectNet is built completely, as shown in Figs 9 and 10.

**Fig 11:**
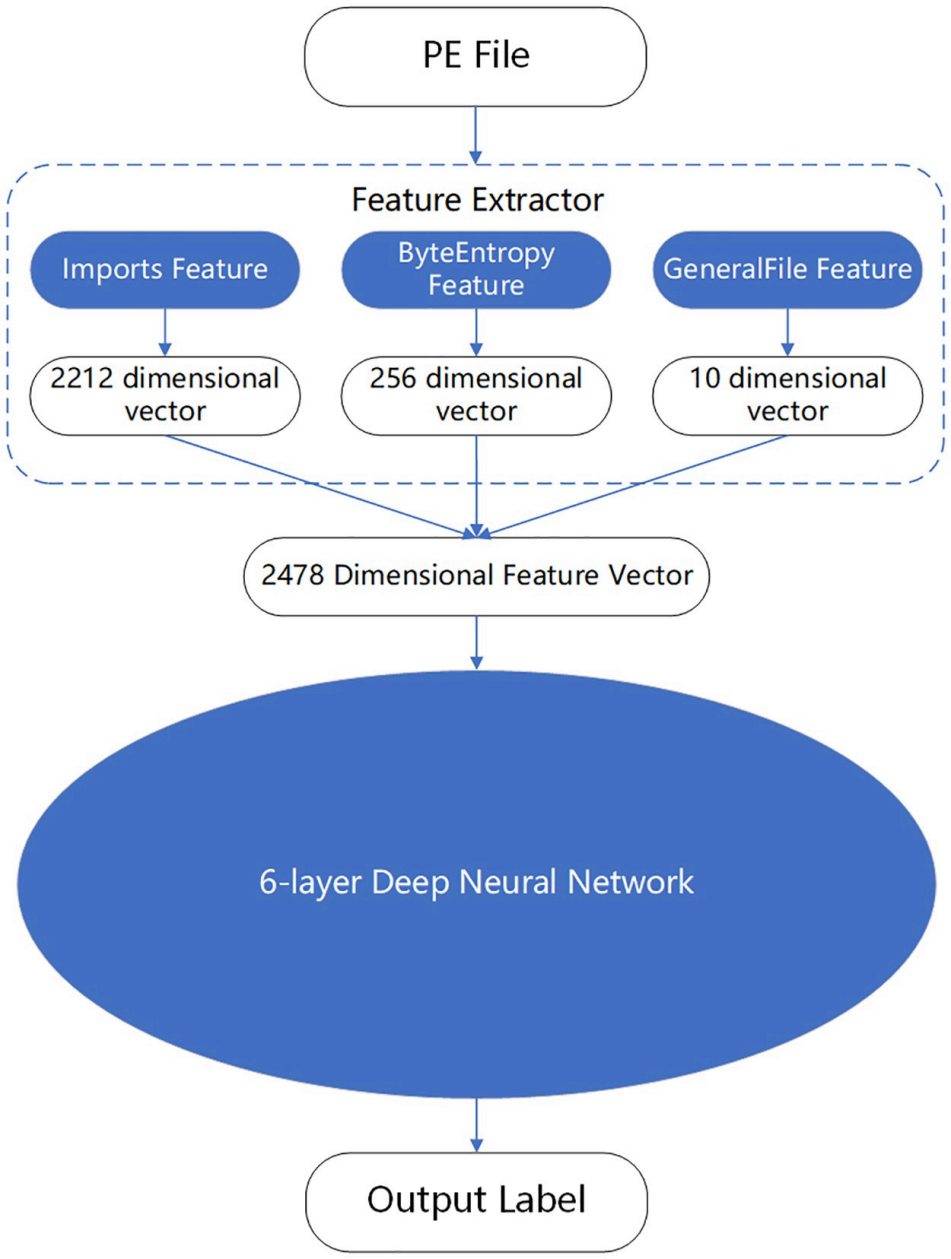
An overview of our DeepDetectNet.

The overview of our DeepDetectNet is shown in Fig 11. According to the experimental results in section, the AUC of this deep learning model can reach 0.989.

### Construction of RLAttackNet

Drawing on the idea of GAN, we need to construct a generation model to attack the detection model (DeepDetectNet). To achieve this goal, our approach is to construct a deep reinforcement learning model.

#### Attack conditions

In [22], the author summarized three attack conditions for malware detection model based on deep learning.

The first condition is the gradient-based direct attack, in which the structure of the detection model is known. However, in general, the attackers do not know the specific structure of the detection model. The second condition is to attack the model that will report scores. The attackers do not know the specific structure of the detection model, but they can continuously input a file and get the corresponding score. The higher the score, the more likely the file is malicious. The third condition is the binary black box attack. The attackers still have no knowledge about the exact structure of the detection model, and they can only input a file to get the corresponding binary value.

To simulate a more realistic attack environment, our method of adversarial sample generation works under the third condition.

#### Modification method of PE files—build the action space

Although the idea of adversarial sample generation comes from GAN, the adversarial samples of PE files need a more specific modification method. We cannot simply add noise like image generation, because that would break the PE file structure, causing the file not to work properly.

In the study of [41], the method of adding import functions to original files was proposed. On this basis, [24] proposed another 9 methods to modify PE files without destroying PE file format and functionality of original files, including:

- add an import function
- modify the name of an existing section
- create a new section
- add some bytes to the free area of the last section
- create a new entry point that immediately jumps to the original entry points
- remove signature information
- modify debugging information
- pack or unpack the file
- modify the header checksum
- add some bytes to the end of PE file

**Fig 12:**
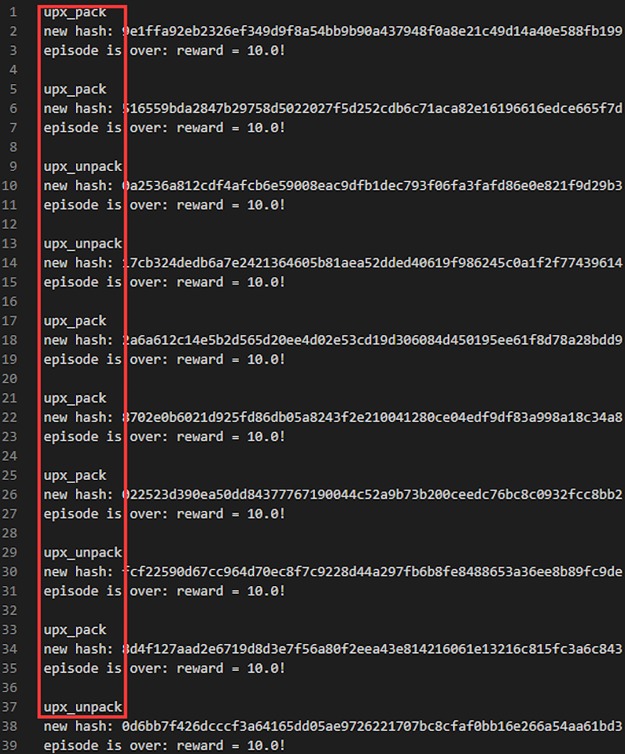
All effective adversarial samples are UPX packed or unpacked. But in general, we don’t regard UPX packing method as a real method of modifying a PE file because it can be easily recognized and resumed by using UPX unpacking method.

However, the implementation of [24] has some randomness. For example, when adding an import function, an unused import function is randomly selected to be added. This leads to some problems with the reinforcement learning model training process. If the *Agent* takes “adding an import function” as the *Action* under a certain *Environment State*, the *reward* may be positive or 0 due to the randomness of the selected import function. If the randomly added function happens to make the original PE file more likely to bypass the detection model, *Q* − *function* will consider it as a good operation that makes the expectation of *Reward* larger when evaluating the *Action* under the *Environment State*. On the contrary, if the randomly added function makes the original PE file more likely to be recognized as a malicious file by the detection model, *Q* − *function* will consider it as a bad operation even if under the same *Environment State*. Of course, *Q* − *function* will feel overwhelmed by this situation, and eventually the *Agent* cannot learn normally! After experiments on the model in [24], as shown in Fig 12, we found that the effective adversarial samples generated by the model are all *UPX* packed or *UPX* unpacked, which cannot be regarded as a real modification of PE file strictly speaking, because a *UPX* packed sample is not only easy to be identified but also easy to be unpacked.

In order to solve this problem, the modification of PE files in this paper no longer contains any random operation. We simplified these operations, which makes the modification of PE files controllable. We will no longer categorize the modification operations, but expand them into many specific operations. Taking “add an import function” operation as an example again, the model in this paper no longer contains the “add an import function randomly” operation. Instead, there are many operations like “add the *CreateFontIndirectW* function in *GDI*32.*dll*”, in which an added function is exactly certain. We carefully selected 200 API functions that do not involve system-sensitive operations like *CreateFontIndirectW*. These API calls are generally not considered malicious calls.

Table 3 shows the *Action* categories and the corresponding number of *Actions* to modify the PE files. It should be noted again that these categories are only for convenience of reading, while the *Agent* will not perceive the existence of categories, because these *Actions* are completely unfolded. In addition to unfolding these *Actions*, this paper also reduces the categories of *Actions* to 6, including “Add an import function”, “Rename a section”, “Add a new section”, “Remove signature”, “Append some random bytes”, and “Remove the debug information”. For “Rename a section”, we have 5 implementations, as shown in Table 4. For “Add a new section”, we implements 10 newly named sections, as shown in Table 5. For “Remove signature”, we can find the Data Directory structure that contains signature information through *IMAGE*_*DIRECTORY*_*ENTRY*_*SECURITY* index in Optional Header’s Data Directories array, and then set *VirtualAddress* and *Size* data fields NULL. For “Append some random bytes”, we look for a section that has free space and then add random number of random bytes within the size of free space. For “Remove the debug information”, like “Remove signature”, we can find the Data Directory structure that contains debug information through *IMAGE*_*DIRECTORY*_*ENTRY*_*DEBUG* index in Optional Header’s Data Directories array, and then set *VirtualAddress* and *Size* data fields NULL.

**Table 3:** Categories of *Action Space*. Note that the categories are only for human to have an intuitive understanding, while the *Agent* will not perceive the existence of categories.

Action Category	Dimension
Add an import function	200
Rename a section	5
Add a new section	10
Remove signature	1
Append some random bytes	1
Remove the debug information	1

**Table 4:** 5 implementations of section rename.

Original Section Name	New Section Name
.text	.mtext
.data	.mdata
.reloc	.mreloc
.rdata	.mrdata
.rsrc	.mrsrc

**Table 5:** 10 implementations of section add.

Added section names
.mpatch0
.mpatch1
.mpatch2
.mpatch3
.mpatch4
.mpatch5
.mpatch7
.mpatch7
.mpatch8
.mpatch9

Finally, the dimension of *Action Space* is 218.

#### Build our agent

For the architecture of reinforcement learning method, we used the DQN (Deep Q-Network) [42] [43] architecture and optimized it with Double DQN method proposed in [44] and Dueling DQN method proposed in [45].

Since DQN belongs to Q-learning in essential, its updating method is similar to Eq (7). The *Q* − *target* in DQN is

$$\begin{array}{c} Y_{t}^{DQN}\equiv R_{t+1}+\gamma \underset{a}{\text{max}}\;Q(S_{t+1},a;\theta_{t}^{-}) \end{array}$$ (11)

where *θ*^−^ represents the integral parameter of Q-network.

In 2016, the research of DeepMind [46] has found the problem of overestimations, which refers to the estimation value of expectation of accumulated *Reward* will be larger than the actual value, always exists.

In order to solve this problem, the method proposed by DeepMind uses two different Q-network, one of which is responsible for selecting the best *Action*, the other of which is responsible for calculating the *Q* − *value*. The *Q* − *target* in Double DQN is expressed by Eq (12).

$$\begin{array}{c} Y_{t}^{DoubleDQN}\equiv R_{t+1}+\gamma Q(S_{t+1},arg\underset{a}{\text{max}}\;Q(S_{t+1},a;\theta_{t});\theta_{t}^{-}) \end{array}$$ (12)

Note that there are two different Q-networks in Eq (12), with parameter *θ* and *θ*^−^ respectively.

Dueling DQN [45] divides the *Q* − *value* into two parts, the *state values*, which mainly evaluates the current *Environment* *State*, and the *action advantages*, which mainly evaluates the goodness of *Actions*. Instead of constructing another Q-network, Dueling DQN only changes the second last layer and the output layer.

The *Q* − *value* in Dueling DQN is

$$\begin{array}{c} Q(s,a;\theta ,\alpha ,\beta )=V(s;\theta ,\beta )+A(s,a;\theta ,\alpha ) \end{array}$$ (13)

where *θ* denotes the parameters of the front layers, while *α* is the parameters only associated with *action advantages* and *β* is the parameters only associated with *state values*. Note that the *state value* function *V*(⋅) has nothing to do with *Actions*. This characteristic is of great significance to our work of adversarial sample generation, because it allows the *Agent* to sense the current malicious degree of the file to be modified at any state.

**Fig 13:**
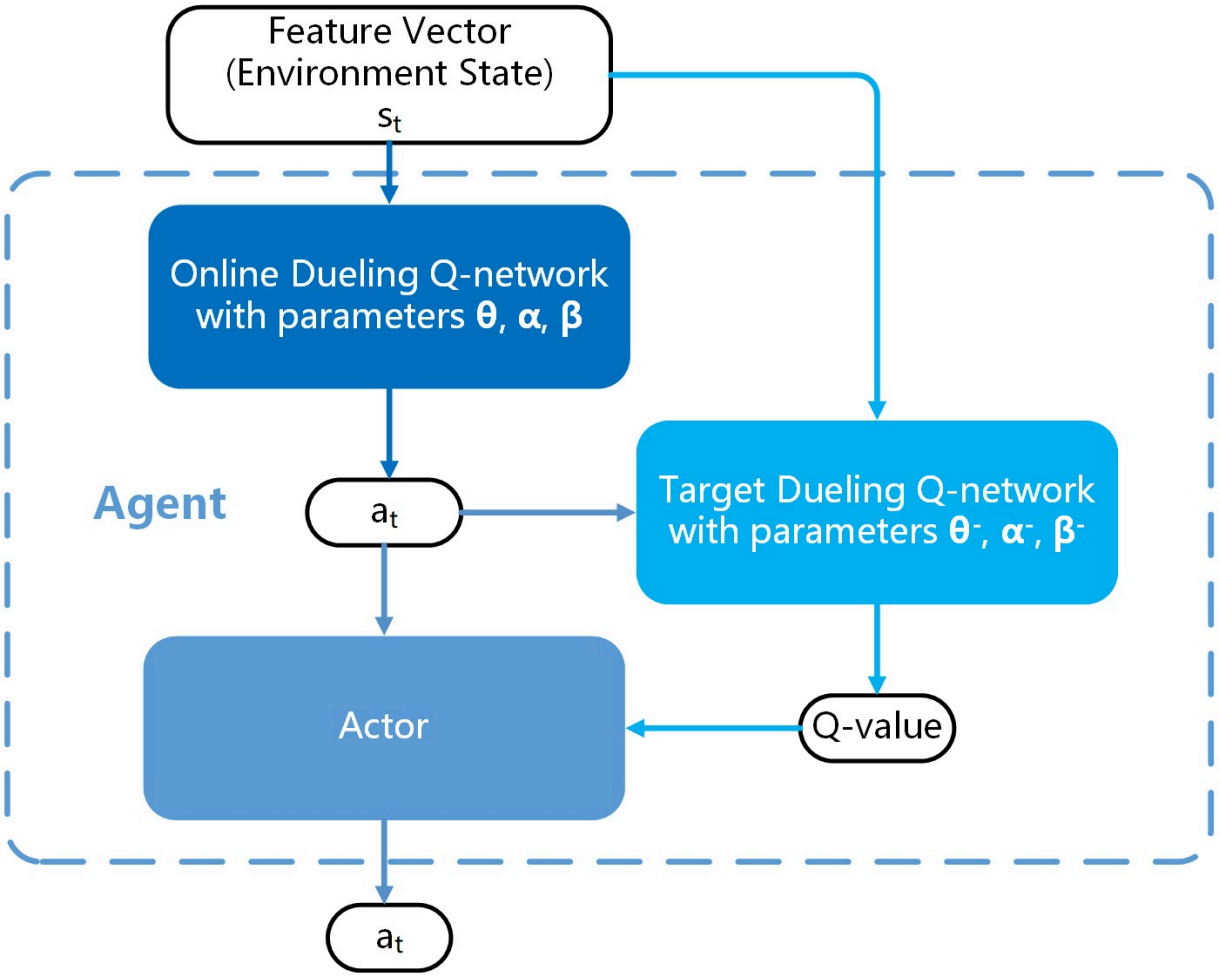
Our double dueling-DQN architecture of *Agent*, where the *Agent* choose the “best” *Action* with the advice of online dueling Q-network and estimate the *Q* − *value* by Target Dueling Q-network.

As described in Double DQN, our *Agent* consists of two networks, one to select an *Action*—the exact method to modify the current PE file, and the other to evaluate the *Q* − *value*. Both of the two networks use the method of Dueling DQN. The architecture of the two networks is the same. The input of the network is the feature vector of the current malicious PE file, which also represents the *Environment State*. More details about feature vector, or *Environment State*, will discuss in section 1. The output layer of the network is a vector made up of action-index corresponding with the *Action Space*. The dimension of the output layer equals 218, referring to the total dimension of *Action Space*. The value of network output is the *Q*−*value*. Our network architecture is shown as Fig 13.

#### Reward

For each episode of training, we have a *MAXTURN* constant that denotes the maximum number of times a malicious PE file can be modified. For each *turn*, the *Agent* select an *Action* to modify the file, and input the file to the detection model built in section.

If the output label is “malicious”, the *reward* is 0, and the *reward* is calculated by Eq (14) when the label is “benign”.

$$\begin{array}{c} r_{t}=k*\frac{MAXTURN}{turn} \end{array}$$ (14)

Considering a significant truth that the order of *Action* to modify the PE file has no effect on the final file, the *reward* of the last *Action* should be an arithmetic average. In Eq (14), both *k* and *MAXTURN* are adjustable parameters. In our experiments, *k* is set to 1 and *MAXTURN* is set to 100. The expected accumulated *Reward* for an episode is calculated as Eq (15).

$$\begin{array}{c} R_{t}=r_{t}+R_{t+1} \end{array}$$ (15)

#### Environment state

For reinforcement learning, the *Agent* needs to observe the current *Environment State* *s*_*t*_, and select the “best” *Action* *a*_*t*_. When it comes to adversarial sample generation, the *Environment State* is the feature vector of a PE file. Since we are going to implement a black box attack, it’s better to use a different feature extraction method from DeepDetectNet. So here, we directly use the feature extraction method of [5], with extracted feature vectors of 2351 dimensions.

#### Adversarial samples generation process

**Fig 14:**
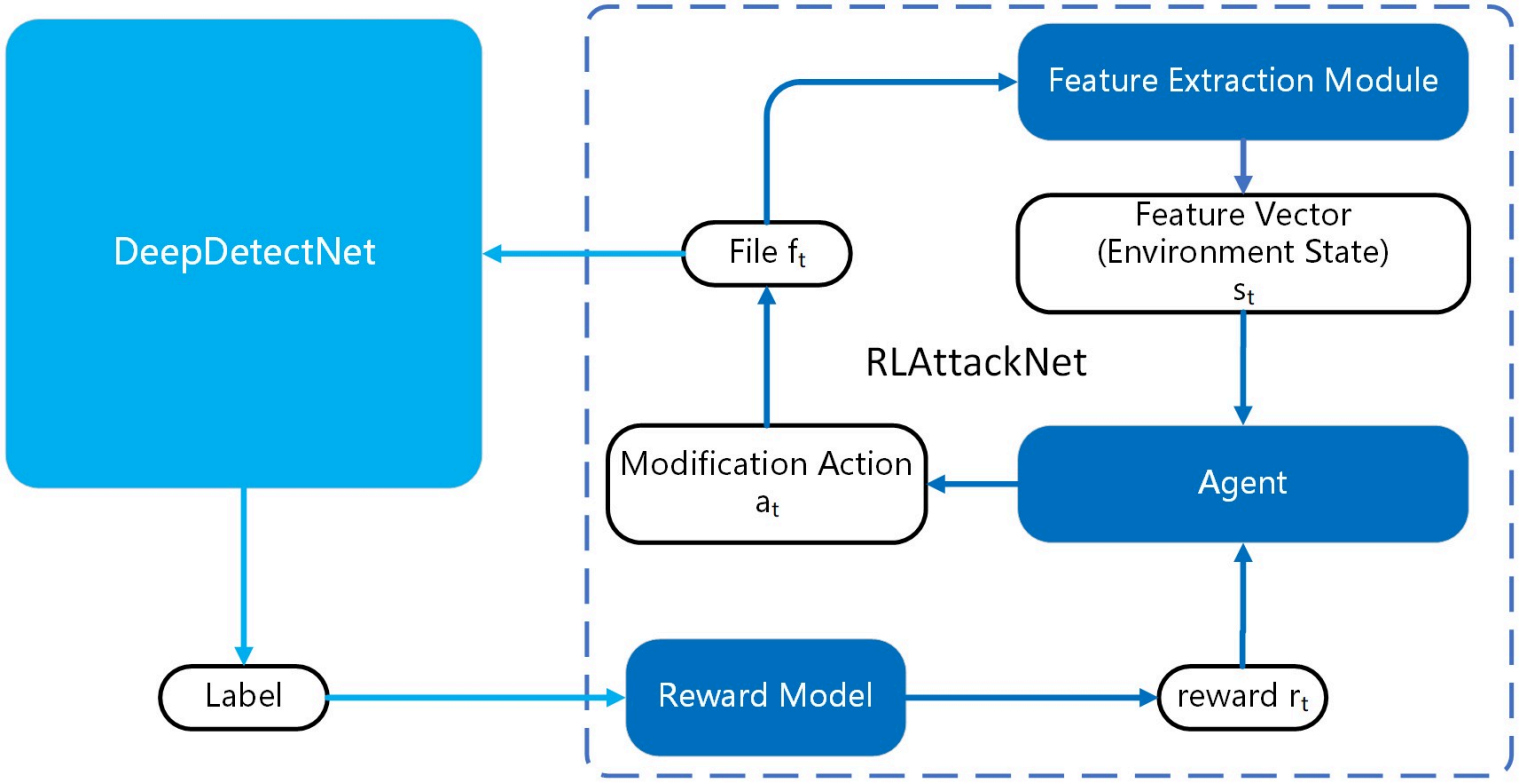
The process of adversarial sample generation. More details are summarized in Algorithm 2.

The process of adversarial sample generation based on reinforcement learning is shown in Fig 14 and the training algorithm of RLAttackNet is summarized in Algorithm 2.

**Algorithm 2** Training Algorithm of RLAttackNet

1: Initialize replay memory *D* to capacity *N*

2: Initialize online dueling network *Q* with random weights *θ*, *α*, *β*

3: Initialize target dueling network *Q*^−^ with random weights *θ*^−^ = *θ*, *α*^−^ = *α*, *β*^−^ = *β*

4: **for** *episode* = 1, *M* **do**

5:  Input the file *f*_*t*_ selected from malicious file sample list

6:  Preprocess *f*_*t*_ with Feature Extraction Module, obtaining the output vector *s*_*t*_ as current Environment State

7:  **for** *t* = 1, *turn* **do**

8:   With probability $P_{s_{t}}$ calculated by Eq (16) select a random action *a*_*t*_ otherwise select *a*_*t*_ = *arg*max_*a*_ *Q*(*s*_*t*_, *a*;*θ*, *α*, *β*)

9:   Modify *f*_*t*_ with action *a*_*t*_ and obtain *f*_*t*+1_. Obtain *s*_*t*+1_ by Feature Extraction Module

10:   Input *f*_*t*+1_ into DeepDetectNet and retrieve the output label, calculating reward *r*_*t*_ using Eq (14)

11:   Store transition (*s*_*t*_, *a*_*t*_, *r*_*t*_, *s*_*t*+1_) in *D*

12:   Sample random minibatch of transitions (*s*_*j*_, *a*_*j*_, *r*_*j*_, *s*_*j*+1_) from *D*

13:   Set

$$R_{j}=\left\{ \begin{array}{ll} r_{j} & \text{if}\;\text{episode}\;\text{stops}\;\text{at}\;\text{step}\;\text{j}+1\\ r_{j}+\gamma \mathbb{Q} & \text{otherwise} \end{array}\right.$$

where $\mathbb{Q}=Q^{-}(s_{j+1},arg{\text{max}}_{a}Q(s_{j+1},a;\theta_{j},\alpha_{j},\beta_{j});\theta_{j}^{-},\alpha_{j}^{-},\beta_{j}^{-})$

14:   Perform RMSProp optimizer on *Loss*_*j*_ calculated by Eq (17) with respect to the network parameters *θ*, *α*, *β*

15:   Every *C* steps reset *θ*^−^ = *θ*, *α*^−^ = *α*, *β*^−^ = *β*

16:   **if** done **then**

17:    break

18:   **end if**

19:  **end for**

20: **end for**

*Line 1∼3 initialization*. In the beginning, we have to initialize our training environment, including our replay memory *D* for experience replay, the weights of online dueling network for action selection, and the weights of target dueling network for Q-value estimation.

*Line 4∼6 obtain the initial environment state*. Traverse all malicious sample files. For each episode, the feature extraction module extracts the feature vector of the input file firstly, the result of which is the initial *Environment State*.

*Line 4∼6 obtain the initial environment state*. The *Agent* selects an *Action* with Boltzmann Q Policy, which selects an *Action* with weighted probabilities $P_{s_{t}}(a)$ that calculated by Eq (16).

$$\begin{array}{c} P_{s_{t}}(a)=\frac{e^{Q_{t}(s_{t},a)}}{\sum_{i}^{m}e^{Q_{t}(s_{t},a)}} \end{array}$$ (16)

*Line 9∼10 interact with DeepDetectNet*. The selected *Action* will modify the PE file and the *Environment State* would have been changed from *s*_*t*_ to *s*_*t*+1_. The modified file will be sent into DeepDetectNet and obtain the result (benign or malicious).

*Line 11∼14 calculate reward and update parameters*. The environment will implement Eq (14) to calculate the *reward* based on the output of DeepDetectNet and feed it back to the *Agent*. To update the parameters of online Q-network, the optimizer RMSProp is performed on *loss* function calculated by Eq (17).

$$\begin{array}{c} Loss_{i}={\left(y_{i}-Q\left(s_{i},a_{i};\theta ,\alpha ,\beta \right)\right)}^{2} \end{array}$$ (17)

*Line 15 Update target Q-network*. Update the parameters of target Q-network every certain steps.

This process cycles *turn* times, while *turn* should be less than *MAXTURN* as mentioned in section. An episode ends when *turn* reaches *MAXTURN*, which denotes the failure, or the output of target detection model shows that the modified PE file is benign, which denotes the success.

## Experiments

### Dataset and environment

**Fig 15:**
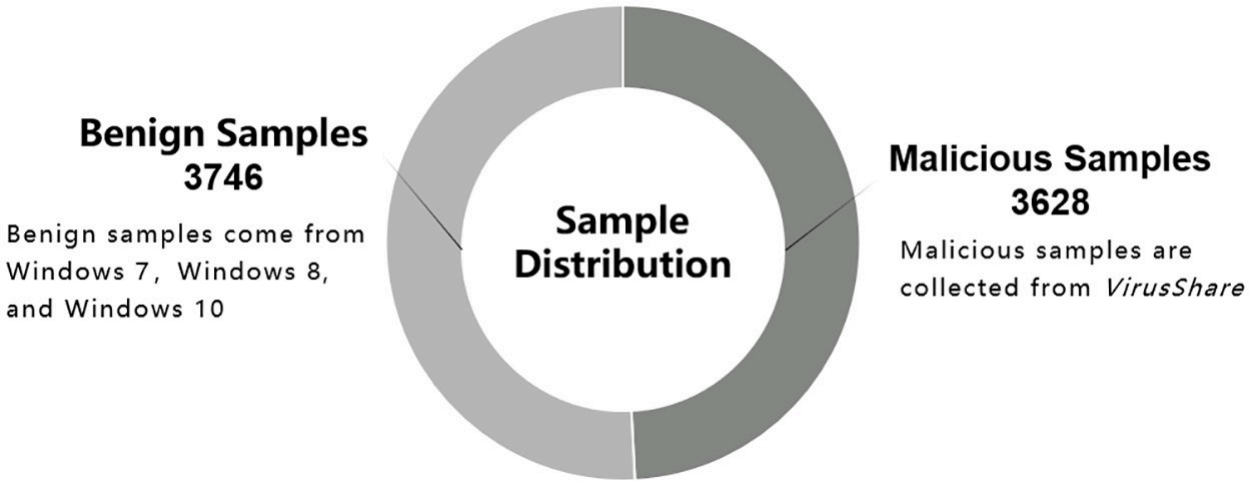
Our sample distribution for training and testing.

In order to prove the effectiveness of our adversarial method to improve detection model, our total number of samples are only 7374, with 3746 benign [47] and 3628 malicious [48], as shown in Fig 15. The benign samples are available at https://doi.org/10.5281/zenodo.3662314, and the malicious samples are available at https://doi.org/10.5281/zenodo.3662293. There’s no risk of malware infection for downloading these samples without running them. But before downloading them, please be sure that you have closed all anti-virus software, including Windows Defender.

Before all experiments are conducted, we need to preprocess our dataset to ensure that all files used for training and testing are PE files. We implement this detection by checking the *e*_*magic* data field in PE Dos Header and the *signature* data field in PE Nt Header. The algorithm of checking valid PE files is summarized in Algorithm 3.

**Algorithm 3** Preprocessing algorithm for checking PE files

1: Input the directory of dataset *Dir*

2: **for** file in *Dir* **do**

3:  Open the file and obtain the file handle *hFile*

4:  Create a file mapping with *hFile* and obtain the handle *hFileMapping*

5:  Obtain the map view of file with *hFileMapping* and obtain the mapping pointer *lpFile*

6:  Read the first chunk of *lpFile* with size of *IMAGE*_*DOS*_*HEADER* and obtain the memory mapping pointer *pDosHeader*

7:  **if** The *e*_*magic* data field of *pDosHeader* is not equal to “MZ” **then**

8:   Remove the file

9:   Continue

10:  **end if**

11:  Read the next chunk of *lpFile* with size of *IMAGE*_*NT*_*HEADERS* and obtain the memory mapping pointer *pNtHeader*

12:  **if** The *signature* data field of *pNtHeader* is not equal to “PE” **then**

13:   Remove the file

14:   Continue

15:  **end if**

16:  Reserve the file

17: **end for**

We implement DeepDetectNet in python 3.7.3 and RLAttackNet in C++ and python 3.7.3. The hardware and software environments for programming and experiments are shown in Table 6.

**Table 6:** Environments for programming and experiments.

Category	Value
CPU	Intel Core i7 7700HQ 2.80GHz
GPU	NVIDIA GeForce GTX 1060 (6G Memory)
RAM	16G DDR4 2400MHz
Operation System	Windows 10 Professional 1903
Programming Language	Python 3.7.3 and C++
GPU Acceleration	CUDA 10.0

### Metrics

For the binary classification problem to be solved by DeepDetectNet in this paper, we use the confusion matrix as shown in Table 7.

$$\begin{array}{c} FPR=\frac{FP}{FP+TN} \end{array}$$ (18)

$$\begin{array}{c} TPR=\frac{TP}{TP+FN} \end{array}$$ (19)

**Table 7:** Confusion matrix.

	Predicted: 0	Predicted: 1
**Actual: 0**	True Negative (TN)	False Positive (FP)
**Actual: 1**	False Negative (FN)	True Positive (TP)

### Procedure of experiments

Firstly, we will use ROC, in which x-axis is *FPR* (defined by Eq (18)) and y-axis is *TPR* (defined by Eq (19)), and AUC, which denotes the area under ROC curve, to verify the effectiveness of the original DeepDetectNet, the result of which will be used as the baseline for later experiments.

Next, we will verify the effectiveness of RLAttackNet. The bypass rate, or the success rate of RLAttackNet, should be greater than bypass rate *P*_*bypass*_(*θ*) of the original DeepDetectNet itself. The bypass rate is calculated by Eq (20), where *θ* represents the parameters of DeepDetectNet. Otherwise, RLAttackNet will be considered invalid, because a malicious sample has the probability of *P*_*bypass*_(*θ*) to be judged as benign. Another significant point we have to pay attention to is the validity of adversarial samples. We will run every adversarial sample that successfully bypass DeepDetectNet in Cuckoo Sandbox [49].

$$\begin{array}{c} P_{bypass}\left(\theta \right)=\frac{FN}{TP+FN} \end{array}$$ (20)

Finally, we will verify that adversarial samples are effective in improving the performance of DeepDetectNet. We need to obtain the new ROC and AUC, and confirm their improvement. What’s more, we have to attack the new DeepDetectNet using RLAttackNet again to confirm that the bypass rate has reduced.

### Performance of original DeepDetectNet

In our experiments, we randomly selected 80% malicious samples and 80% benign samples as the training set, and the remaining 20% as the testing set.

Since the total number of samples is small, 20 epochs of training is enough to achieve a good result. For each epoch, the number of a batch is set 100. While the experiments were conducted under NVDIA GEFORCE GTX 1060 with CUDA 10.0, every epoch only took about 3s 344ms on average.

**Fig 16:**
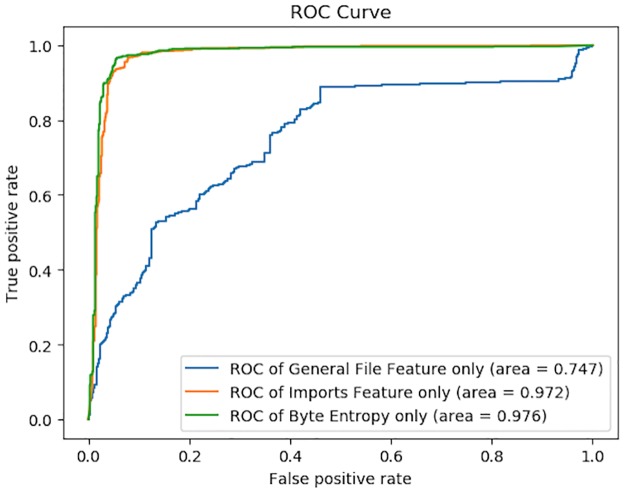
Performances of original DeepDetectNet with only single type of feature. As shown in this figure, when training the DeepDetectNet with General File Feature individually, the AUC value can reach 0.747. When training the DeepDetectNet with Imports Feature individually, the AUC value can reach 0.972. When training the DeepDetectNet with Byte Entropy Feature individually, the AUC value can reach 0.976. Since all of the AUC results are significantly greater than 0.5, all of these features are effective.

To verify the validity of features we selected, we firstly took each type of feature as the input vector and trained the corresponding deep neural network separately. If the finally AUC value is significantly greater than 0.5, we think the single type of feature is effective. The ROC curves and AUC values of each model with single type of feature are shown in Fig 16.

**Fig 17:**
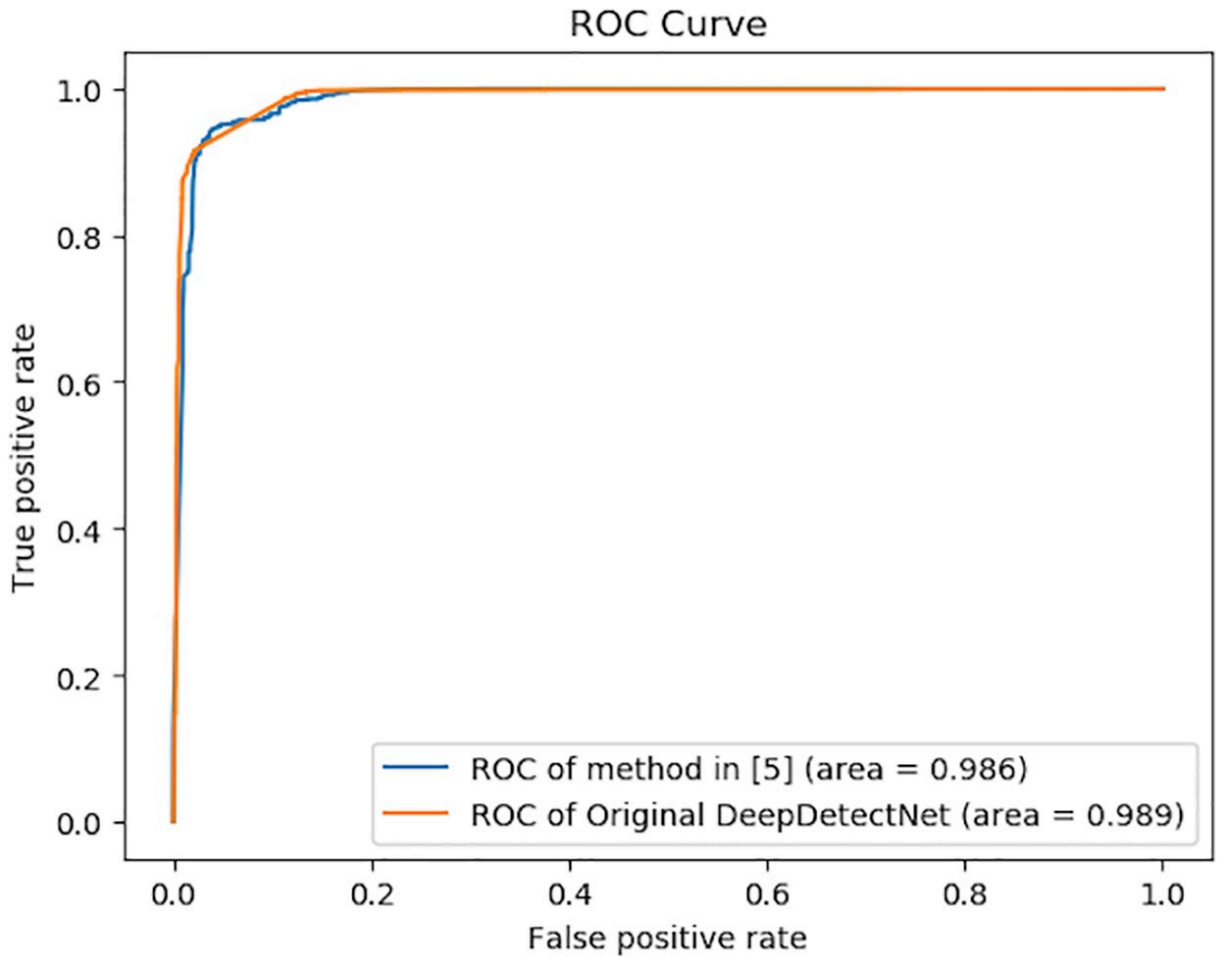
Performances of original DeepDetectNet and method in [5]. Having combined all of these three types of feature, our DeepDetectNet performs well with the AUC value 0.989, slightly greater than 0.986—the AUC result of method in [5]. The comparison shows that our DeepDetectNet is a great deep learning model.

The results of original DeepDetectNet with combined features we proposed above and the method proposed in [5] are shown in Fig 17. The results show that the AUC value of DeepDetectNet is slightly higher than the method proposed in [5], which means our DeepDetectNet is effective and available. This result would be used as the baseline for later experiments. Among the total of 1475 testing samples, there are 726 malicious ones with label 1. Among these testing samples whose actual label is 1, our original DeepDetectNet predicted 712 malicious samples correctly, which means, the *TPR* of original DeepDetectNet can reach 98.07%. Therefore, the original bypass rate *P*_*bypass*_(*θ*_0_) is equal to 1.93%. Original AUC can reach 0.989.

### Performance of RLAttackNet

Before this part of experiments, we have to do some preparations. We deployed the environment of Cuckoo Sandbox [49], which can provide a detailed report, outlining the behavior of the input PE file when executed inside a realistic but isolated environment. With the help of Cuckoo Sandbox, we can make sure that an *Action* will not corrupt the sample’s PE format. What’s more, we also need to ensure the function of the modified file will not be changed. So we used IDA Pro [50] to demonstrate a sample’s function call flow chart. If the function call flow chart of original sample and modified sample are the same, we can conclude that *Actions* have not affected the function of the sample.

We write a dynamic link library in C++, implementing all methods in Table 3, which is adapted with 32-bit and 64-bit PE file formats. In our main python programs, we use CDLL library to call these interfaces.

To sum up, after each successful episode, in which an adversarial sample that can bypass the original DeepDetectNet was generated, we save the adversarial sample and throw it into Cuckoo Sandbox and IDA Pro. Fortunately, as shown in experiments, the structure and function of each adversarial sample remained fine.

**Fig 18:**
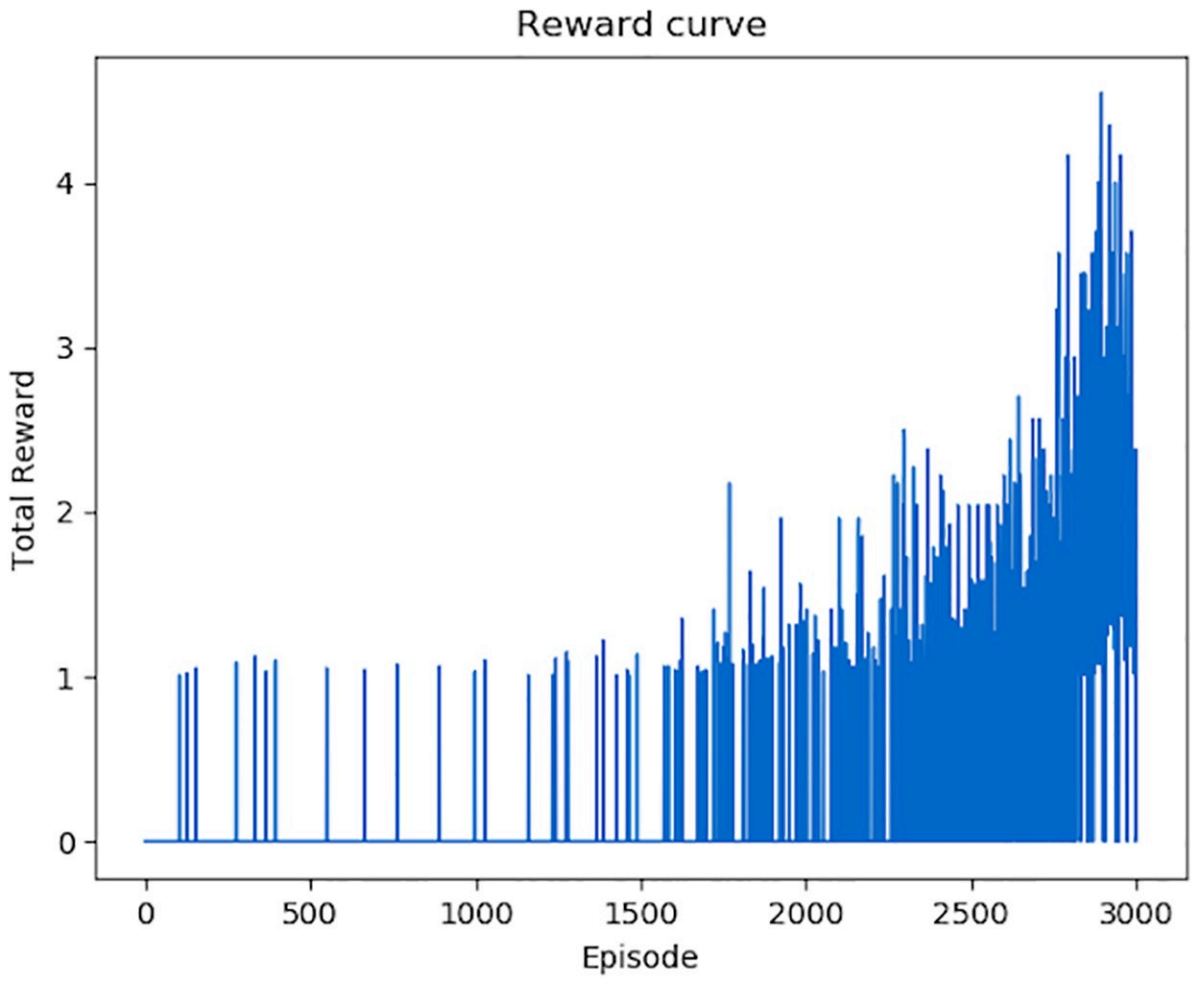
Total *Rewards* for each episode. In the first 1500 modification episodes, approximate 150000 *Actions*, the *Rewards* for each episode is extremely low, most are 0. With the increasing time of episodes, the *Rewards* for each episode is becoming more and more.

**Fig 19:**
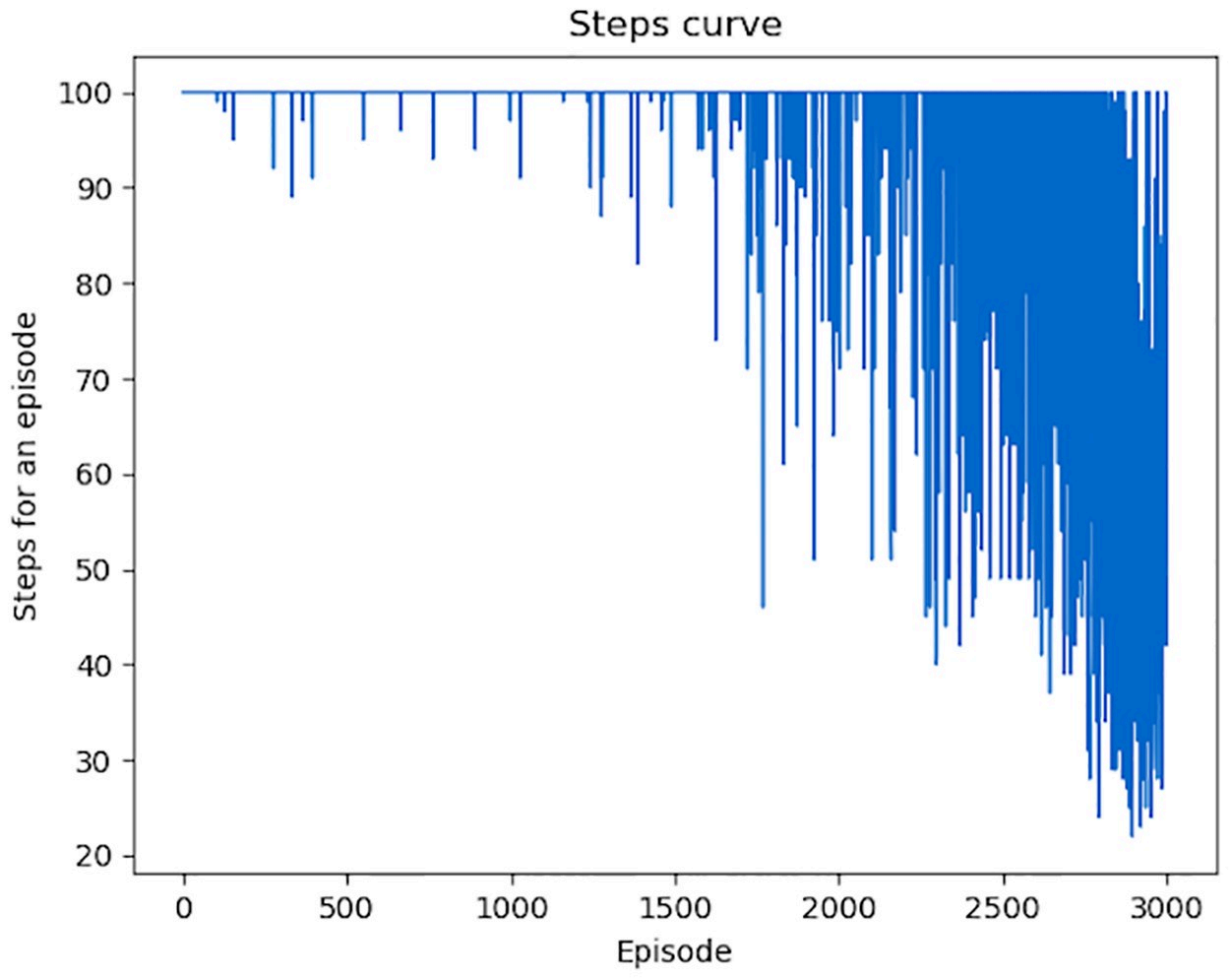
Total steps for each episode. With the increasing time of episodes, total steps, equal to the times of *Actions*, for each episode, gains a significant decline.

The variation of total *Rewards* for each episode is shown in Fig 18. An episode means a set of modifications (*Actions*) on one malicious PE file. We conducted 3000 episodes to show the variation trend of *Rewards* obtained by each episode. At the beginning, the *Agent* can only generates some successful adversarial samples by accident, and in most episodes, the total *Rewards* is 0. With the increasing number of episodes, the average *Rewards* for an episode is also increasing. The variation of total steps for each episode is shown in Fig 19. Apparently, the number of *Actions* performed by the *Agent* is becoming fewer and fewer.

Among the 3000 episodes, the *Agent* generated 574 successful adversarial samples which can bypass DeepDetectNet. The bypass rate is 19.13%, far more than *P*_*bypass*_(*θ*_0_), so the attack process of RLAttackNet is valid.

### Performance of retrained DeepDetectNet

We added the 574 adversarial samples generated by RLAttackNet into the data set and retrained our DeepDetectNet. The performance of retrained DeepDetectNet has been improved.

**Fig 20:**
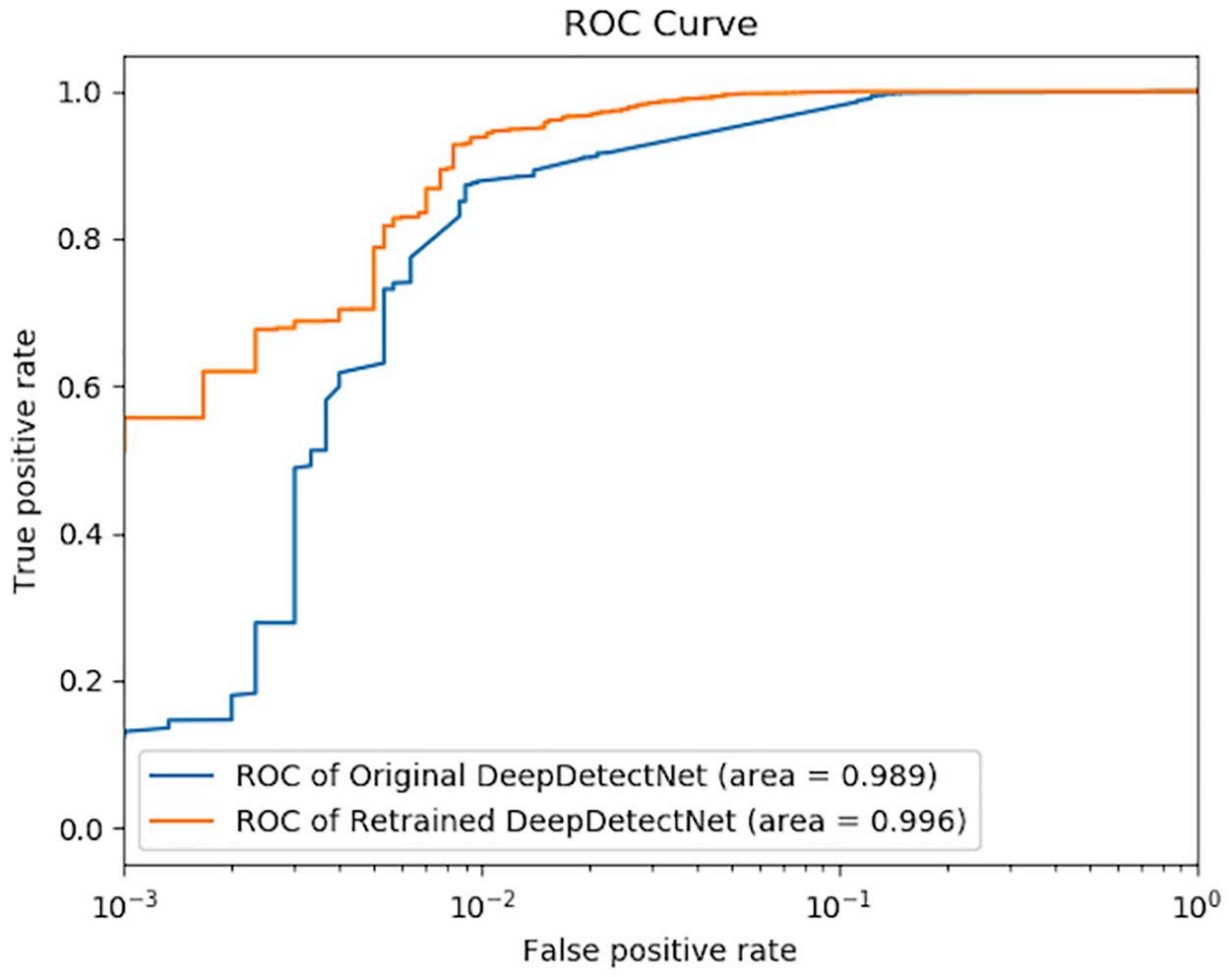
A comparison between original DeepDetectNet and retrained DeepDetectNet. The AUC value improved from 0.989 to 0.996.

The ROC curves of retrained DeepDetectNet and original DeepDetectNet are shown in Fig 20. Among the new 841 malicious samples with actual label 1, the retrained DeepDetectNet can recognize 834 of them correctly, with bypass rate *P*_*bypass*_(*θ*_1_) reduced to 0.83%. The comparison of bypass rate between original and retrained DeepDetectNet is shown in Table 8.

**Table 8:** Comparison between original and retrained DeepDetectNet when each type of feature is used as input vectors separately.

	Original Bypass Rate	Retrained Bypass Rate
*P*_*bypass*_(*θ*)	1.93%	0.83%
**Attacked by RLAttackNet**	19.13%	3.1%

**Fig 21:**
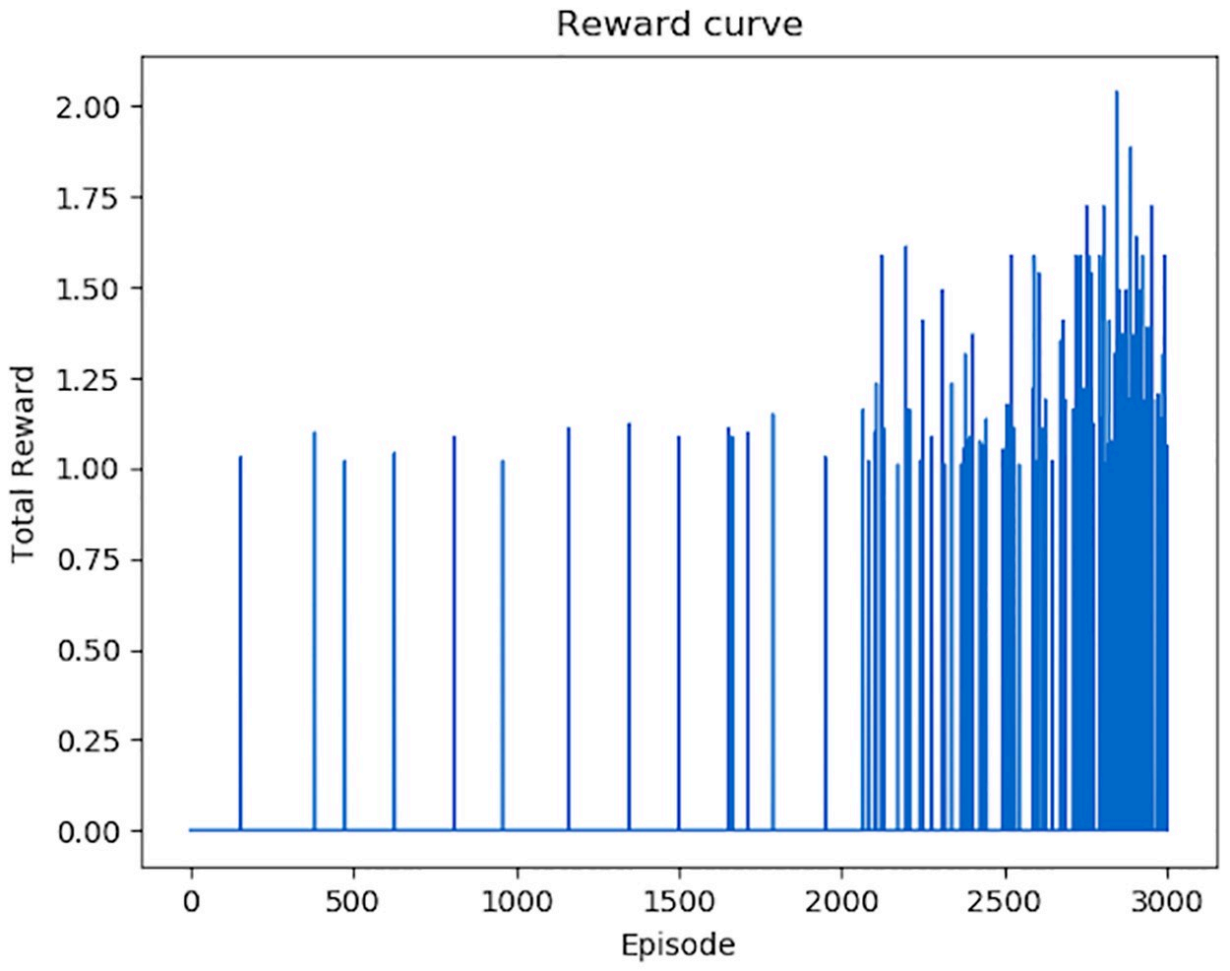
Total *Rewards* for each episode when attacking retrained DeepDetectNet. Compared with Fig 18, both the average value of total *Rewards*, and the count of episodes that total *Rewards* greater than 0, significantly declined.

**Fig 22:**
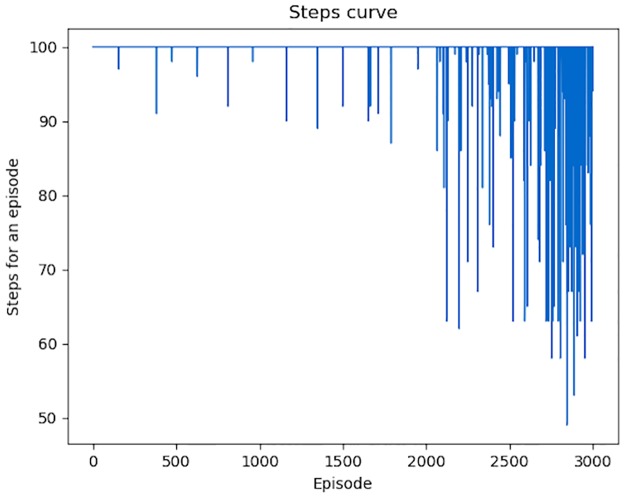
Total steps for each episode when attacking retrained DeepDetectNet. Compared with Fig 19, both the average step value, and the minimum steps in an episode, significantly increased.

What’s more, when we attack the retrained DeepDetectNet with RLAttackNet, also 3000 episodes conducted, the number of successful adversarial samples is only 93. The variation of total *Rewards* and steps are shown in Figs 21 and 22. New bypass rate is reduced to 3.1%, far less than 19.13%—the bypass rate of original DeepDetectNet.

In addition, each single type of feature gains a better performance. When these types of features are used as input vectors of DeepDetectNet, the comparison between original and retrained DeepDetectNet is shown in Table 9.

**Table 9:** Comparison between original and retrained DeepDetectNet when each type of feature is used as input vectors separately.

	Original AUC	Retrained AUC
**Imports Feature**	0.972	0.992
**BytesEntropy Feature**	0.977	0.986
**GeneralFile Feature**	0.747	0.782
**All Features**	0.989	0.996

From the above comparisons, it can be seen that retrained DeepDetectNet has been significantly improved in performance.

## Conclusion

In this paper, drawing on the idea of GAN, we present a novel adversarial method in the field of static PE malware detection. We construct an adversarial sample generation model based on deep reinforcement learning, called RLAttackNet, to improve static PE malware detection model based on deep learning, called DeepDetectNet. An attack and defense scenario for static PE malware detection is presented in our work.

First, having investigated recent years’ research on static malware detection model based on deep learning, we derive two main conclusions: 1) in the field of static PE malware detection, deep learning model has more effectiveness than traditional machine learning method with shadow neural network; 2) in the work of feature extraction for static PE malware detection, the feature engineering-based method has a better performance than methods based byte sequences or opcode sequences. Based on these two points, this paper mainly carried out the following two aspects in the construction of static PE malware detection model: 1) accomplished feature extraction work based on feature engineering method. In this paper, we have made an improvement on feature engineering method by selecting three categories of features; 2) constructed the deep neural network structure suitable for PE file static detection. Hence, a static PE malware detection model based on deep learning called DeepDetectNet is constructed. The experimental result shows that the original AUC of DeepDetectNet can reach 0.989.

Second, similar to the picture recognition model, the static malware detection model based on deep learning is also vulnerable to the interference of adversarial samples. In order to find the defects of DeepDetectNet, and considering the practical difficulties in direct mathematical derivation, we need to attack DeepDetectNet by generating adversarial samples. Since the existing malware adversarial sample generation method is not universal and low in generation efficiency due to the need for human control and difficulty in maintaining a normal file format, we proposed a novel method of automatic adversarial samples generation based on deep reinforcement learning. We construct the adversarial generation model called RLAttackNet based on Double DQN and Dueling DQN architecture with an extremely practical Action Space, in which actions can modify a PE file without breaking the file’s structure and functionality. The constructed RLAttackNet is deployed to attack DeepDetectNet, generating adversarial samples that can bypass DeepDetectNet. The experimental results show that the success attack rate can reach 19.13

Finally, to reinforce the defects of original DeepDetectNet, we add the generated adversarial samples to our dataset and retrain DeepDetectNet. The experimental results show that the AUC value improved from 0.989 to 0.996 and *P*_*bypass*_(*θ*) dropped from 1.93% to 0.83%. Meanwhile, the success attack rate dropped from 19.13% to 3.1%.

In the future, we can use more evaluation approaches to validate the results, such as k-cross-validation approach. On the other hand, for a deep learning model, there are many hyper-parameters, including learning rate, epoch times, and so on, that may significantly improve the effectiveness of a model. In this work, we use the random search method, but some hyper-parameter optimization methods that can find optimal hyper-parameters have been proposed. For example, Dong et al. [51] proposed a novel hyper-parameter optimization method for a given sequence using an action-prediction network leveraged on Continuous Deep Q-Learning. We will concentrate more on hyper-parameter optimization methods in the deep learning model in the future.
